# Robust Heterogeneity Adjustment for Gaussian Graphical Model With Latent Variables

**DOI:** 10.1002/sim.70571

**Published:** 2026-05

**Authors:** Linxi Li, Rong Li, Shuangge Ma, Qingzhao Zhang

**Affiliations:** 1 Department of Statistics and Data Science, School of Economics, Xiamen University, Fujian, China; 2 Center for Applied Statistics and School of Statistics, Renmin University of China, Beijing, China; 3 Institute of Health Data Science, Renmin University of China, Beijing, China; 4 Department of Biostatistics, Yale School of Public Health, Connecticut, USA; 5 The Wang Yanan Institute for Studies in Economics, Xiamen University, Fujian, China

**Keywords:** Gaussian graphical models, gene expression network, heterogeneity, latent variables, robust

## Abstract

Graphical models serve as fundamental tools for encoding conditional dependence structures in multivariate biological data, with latent variable Gaussian graphical models playing a pivotal role in capturing complex dependencies in the presence of unobserved confounding variables. However, practical implementations often face two critical challenges: systematic heterogeneity arising from unobserved subpopulations (e.g., tumor subtypes, cell clusters, or patient stratifications) and outliers (e.g., technical artifacts or rare phenotypic variations), both of which can substantially distort the underlying network structure. To address these issues, we extend the latent variable Gaussian graphical model by integrating a mixture model, proposing a robust framework tailored for data heterogeneity. The proposed method can simultaneously achieve network structure estimation (after removing shared effects from latent variables), outlier detection, and subgroup membership identification. An effective computational algorithm is developed. Extensive experimental evaluations demonstrate that the proposed method offers a reliable graphical estimate in the presence of heterogeneity, maintaining robustness even against a significant proportion of outliers. The heterogeneity analysis of a breast cancer dataset further illustrates the practical applicability of the proposed approach and its sound biological implications.

## 1 | Introduction

Graphical models are powerful tools for representing conditional dependencies among variables across various fields of study. Among them, the Gaussian graphical model (GGM) is commonly used to estimate network structures. In many practical applications, not all relevant variables are observed—some are latent and unobserved. Such latent variables can induce “indirect” interconnections among the observed ones, leading to a dense network. For example, when constructing the gene network, latent genomic regulators can influence the interconnections among gene expressions. To address this issue, Chandrasekaran et al. [1] propose the latent variable Gaussian graphical model (LVGGM) to learn the network structure with latent effects removed. Subsequent research expands this framework along multiple frontiers [2–5].

**FIGURE 1 |:**
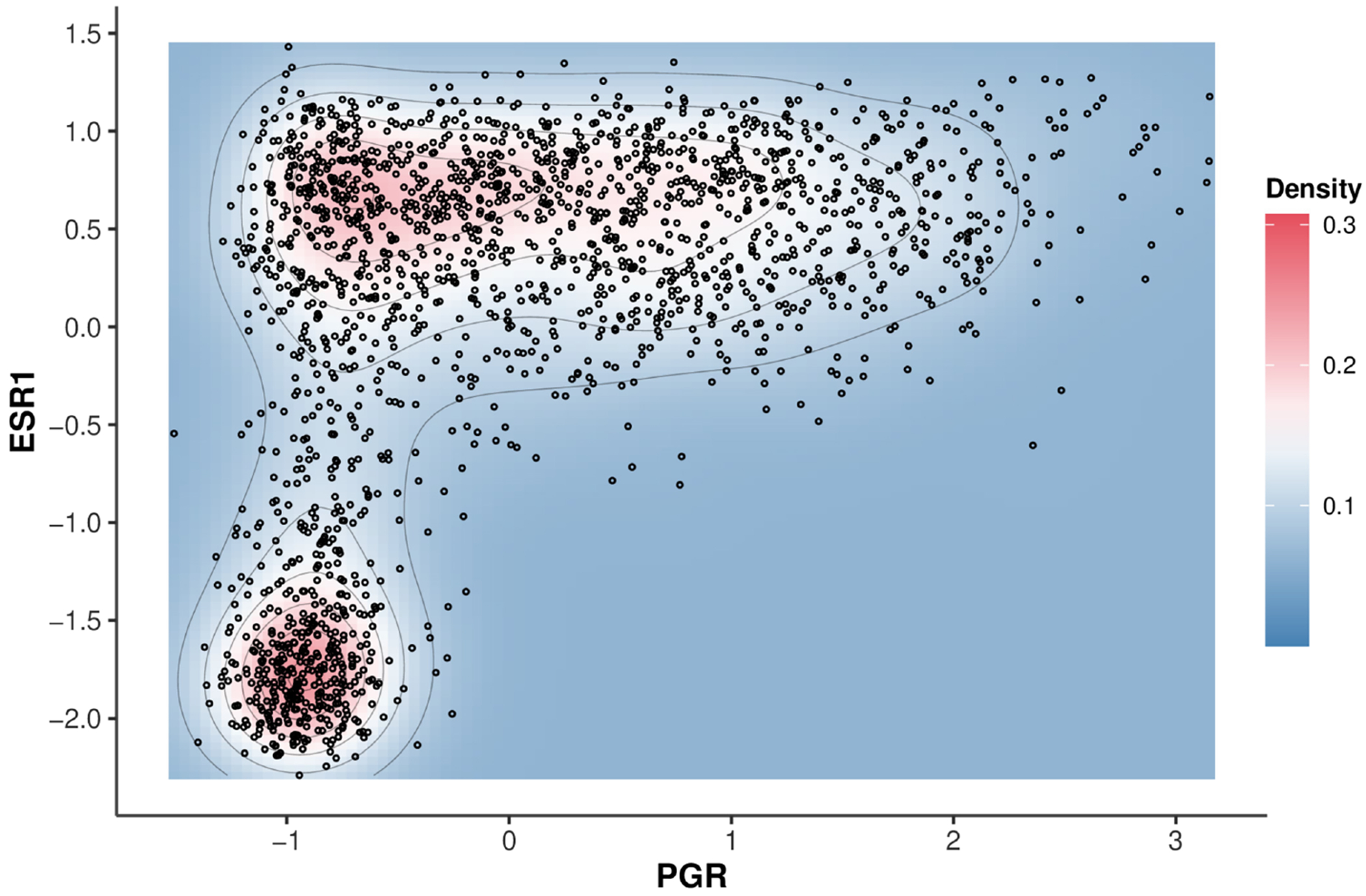
Two-dimensional density plot of PGR and ESR1 gene expression in METABRIC breast cancer data.

There are several issues that make the estimation of network structure in the context of gene expression data particularly challenging. The first one is molecular heterogeneity arising from cellular and tissue-level variation, especially for cancer patients. For example, when analyzing breast cancer data from the METABRIC study, it is intuitive to consider cancer subtypes, as breast cancer is highly molecularly heterogeneous [6]. Figure 1 shows a scatter plot of ESR1 and PGR gene expression with the density contours. The plot exhibits two distinct peaks in red, indicating the presence of at least two subgroups with distinct gene expression patterns. As such, it is impossible to achieve equal means across subgroups and lead to mean zero by centralization. Therefore, it is crucial to consider the heterogeneous mean component in network structure estimation. Another challenge is the presence of outliers, as illustrated by the observations located at the periphery of the density in Figure 1. These outliers could bias dependency estimates and lead to wrong analysis in graphical modeling [7–9]. In addition, the outliers are spread over all data samples, influencing the identification of subgroups. Therefore, effective network estimation requires careful consideration of both data heterogeneity and outliers. Beyond their impact on network structure, identifying heterogeneity and outliers is of independent interest in molecular data analysis.

In the study of graphical models with heterogeneity, most existing approaches belong to two families. The first considers that the heterogeneity pattern (i.e., subgroup membership) is known. Examples include Danaher et al. [10]; Guo et al. [11], which use integrated analysis to achieve the improvement. A special category within this family is heterogeneous graphical models depending on covariates, including either covariate-dependent mean structures or network structures [12–18]. The second family is based on unknown heterogeneity in samples. GGMs built upon mixture distributions are proposed to achieve simultaneous clustering and cluster-specific network estimates [19–22]. Both families contribute to incorporating population heterogeneity into graphical modeling, whereas they do not consider the effects of latent variables or outliers.

To address the outliers, a body of literature focuses on providing more robust estimates for the precision matrix in graphical models [8, 23 – 26]. However, these methods are not good at dealing with heterogeneity. Robustness in heterogeneity analysis has been explored in the framework of the mixture model, through robust distributions or estimates. Examples include McLachlan and Peel [27] and Qin and Priebe [28]. Additionally, inspired by the addition of a uniform noise component to Gaussian mixture [29], Coretto and Hennig [30] propose the optimally tuned robust improper maximum likelihood estimator (OTRIMLE) for robust clustering, which can effectively identify outliers. To the best of our knowledge, there remains a methodological gap in heterogeneous graphical models that are robust against outliers, let alone accounting for latent variables.

In this study, our goal is to develop a new graphical model-based method to enhance the estimation of network structure among variables. Building on existing literature, the proposed method advances in multiple important aspects. First, the proposed method is designed to mitigate contamination from heterogeneity and outliers to obtain a more robust and effective network estimate. We adopt the second family with unknown subgroups and focus on the distinct mean vectors across subgroups—note that the proposed strategy can be straightforwardly extended to distinct precision matrices, albeit with slight modifications in computation. Second, with the robust mixture model we developed, itis capable of simultaneously identifying subgroup membership and detecting outliers, both of which are equally important and enhance its applicability to real-world analysis. Third, benefiting from the incorporation of latent variables in the graphical model, it can identify “direct” interconnections removing the shared effects from latent variables and recover the “core” network structure. Last but not least, as partly demonstrated by our data analysis, this study can provide a practical tool for comprehensively understanding the mechanisms and heterogeneity of complex diseases. Although applied here to gene expression data, the method has broader applicability. It can be readily extended to other omics modalities, including transcriptomic platforms, proteomic profiles, metabolic signatures, as well as neuroimaging applications, such as functional brain network analysis.

The rest of the paper is organized as follows. In Section 2, the proposed robust heterogeneity-adjusted latent variable Gaussian graphical model is introduced. Section 3 presents simulation studies, where the performance of the proposed method is evaluated and compared to relevant alternative methods. A breast cancer data analysis from the METABRIC database is presented in Section 4. Concluding remarks are provided in Section 5. Additional numerical and application results are provided in Appendices A and B.

## 2 | Methodology

### 2.1 | Latent Variable Gaussian Graphical Model

Considering the presence of latent variables in the Gaussian graphical model, let ***x*** represent the p observed variables and z represent the r latent variables (r<p). Assume that the joint distribution of the (p+r)-dimensional random vector ${\left(x^{⊤},z^{⊤}\right)}^{⊤}$ follows a Gaussian distribution with covariance matrix $\tilde{\Sigma }$ and precision matrix $\tilde{\Theta }≔{\tilde{\Sigma }}^{-1}$. In the full model, the submatrices of $\tilde{\Theta }$ specify the conditional dependencies among the observed variables, between the observed and latent variables, and among the latent variables, respectively. Upon marginalizing over the latent variables, Chandrasekaran et al. [1] show that the distribution of the observed variables x remains Gaussian, with the observed covariance matrix given by $\Sigma ={\tilde{\Sigma }}_{1:p,1:p}$, where ${\tilde{\Sigma }}_{1:p,1:p}$ denotes the submatrix consisting of the first p rows and columns. The marginal precision matrix of $x,\Theta ≔\Sigma^{-1}$ is given by Schur complement:

$$\Theta =\underset{S}{\underbrace{{\tilde{\Theta }}_{1:p,1:p}}}-\underset{L}{\underbrace{{\tilde{\Theta }}_{1:p,(p+1):(p+r)}{\tilde{\Theta }}_{\left(p+1):(p+r),(p+1):(p+r\right)}^{-1}{\tilde{\Theta }}_{(p+1):(p+r),1:p}}}.$$

Denote $S≔{\tilde{\Theta }}_{1:p,1:p}$ and $L≔{\tilde{\Theta }}_{1:p,(p+1):(p+r)}{\tilde{\Theta }}_{\left(p+1):(p+r),(p+1):(p+r\right)}^{-1}{\tilde{\Theta }}_{(p+1):(p+r),1:p}$. The matrix L is low-rank when the number of latent variables is small relative to the number of observed ones. Therefore, the marginal precision matrix is generally non-sparse due to this additional low-rank component.

### 2.2 | The Proposed Estimator

For each observation i=1,…,n, a p-dimensional vector $x_{i}={\left(x_{i1},\ldots ,x_{ip}\right)}^{⊤}$ is measured, and the n observations are independent. We assume that the n subjects belong to K-th subgroup. In the k-th subgroup, $k=1,\ldots ,K,x_{i}∼𝒩\left(\mu_{k},\Sigma \right)$ with precision matrix Θ, encoding the interconnections between variables. Due to the existence of latent variables, the precision matrix is parameterized as Θ=S−L following LVGGM, where S is a sparse matrix representing the direct interconnections among the observed variables, and L is a low-rank matrix (with rank r<p) representing the effect of marginalization over the latent variables. We focus on heterogeneous latent variable graphical models with unknown labels. Assume the subjects follow a mixture model with the density function:

(1) $$f(x)=\sum_{k=1}^{K}\pi_{k}f_{k}\left(x;\mu_{k},{\left(S-L\right)}^{-1}\right),$$

where $f_{k}$ denotes the probability density function of the multivariate Gaussian distribution $𝒩\left(\mu_{k},(S-L{)}^{-1}\right)$, that is,

$$f_{k}\left(x;\mu_{k},(S-L{)}^{-1}\right)=(2\pi {)}^{-p/2}|\Sigma {|}^{-1/2}exp\left(-\frac{1}{2}{\left(x-\mu_{k}\right)}^{⊤}(S-L{)}^{-1}\left(x-\mu_{k}\right)\right).$$

This distribution accommodates both the heterogeneity induced by subgroup-specific mean values and the underlying network structure through a sparse plus low-rank decomposition. Notably, we prioritize subgroup-specific mean vectors having a common precision matrix, following the discussion in McNicholas and Murphy [31]. The proposed strategy allows straightforward extension to subgroup-specific precision matrices with $\Theta_{k}=S_{k}-L_{k}$ and the mixture density $\sum_{k=1}^{K}\pi_{k}f_{k}\left(x;\mu_{k},{\left(S_{k}-L_{k}\right)}^{-1}\right)$. Considering the model complexity, a compromise is to allow all subgroups to share a common low-rank component **L** but subgroup-specific sparse components $S_{k}$, that is, $\Theta_{k}=S_{k}-L$—captures shared latent effects but different direct interconnections after removing the effects from latent variables.

When the data are contaminated by outliers, including the observations from the population other than the target ones, and corrupted due to either human error or instrument malfunction, we modify the mixture density function (1) for robustness. We introduce an improper constant density parameter 0≤δ<1 that accounts for uniformly distributed contamination across the data domain, calling it the noise component. This noise component effectively models outliers as a uniform distribution spanning the entire observation space, reducing the influence of outlier samples while preserving the major distribution characteristics of the heterogeneous data. Overall, we propose the following penalized objective function:

(2) $$ℒ\left(\delta ,\mu_{\left(K\right)},S,L,\pi |X\right)=\frac{1}{n}\sum_{i=1}^{n}\log\left(\pi_{0}\delta +\sum_{k=1}^{K}\pi_{k}f_{k}\left(x_{i};\mu_{k},{\left(S-L\right)}^{-1}\right)\right)\;-\sum_{i\neq j}p\left(\left|S_{ij}\right|;\lambda_{1}\right)-\sum_{j=1}^{p}p(\sigma_{j}\left(L\right);\lambda_{2}),\;\text{subject to}\;S-L≻0,L≽0,$$

where $X={\left(x_{1},\ldots ,x_{n}\right)}^{⊤},\mu_{\left(K\right)}=\left(\mu_{1},\ldots ,\mu_{K}\right)$, and $\pi =\left(\pi_{0},\pi_{1},\ldots ,\pi_{K}\right)$ is mixture proportions with $\pi_{k}\in [0,1]$ for k=0,1,2,…,K and $\sum_{k=0}^{K}\pi_{k}=1$. Here, $S_{ij}$ denotes the (i,j)-th entry of matrix $S,\sigma_{j}(L)$ represents the j-th eigenvalue of L, and p(⋅;λ) is a non-convex penalty function with tuning parameter λ>0. The constraint S−L≽0 implies that the matrix S−L is positive definite, and L⩾0 implies that L is positive semi-definite. These constraints are necessary to prevent Θ from becoming indefinite and to ensure a well-defined Gaussian likelihood and valid covariance structure. Additionally, for identifiability, L should also be incoherent, that is, its row/column spaces are spread uniformly across all coordinates instead of being concentrated in a few.

The two penalties in (2) correspond to the conditional sparsity and low-rank structure inherent in the LVGGM, respectively. In the numerical studies of this paper, we employ the minimax concave penalty (MCP) function proposed by Zhang [32], and other penalties such as smoothly clipped absolute deviation (SCAD) [33] are also applicable. We can obtain the estimate by $\left(\overset{ˆ}{\delta },{\overset{ˆ}{\mu }}_{\left(K\right)},\hat{S},\hat{L},\hat{\pi }\right)=arg\;max\;ℒ\left(\delta ,\mu_{\left(K\right)},S,L,\pi |X\right)$, which is robust against outliers and adjusted for heterogeneity. Additionally, the proposed method allows incorporating a penalty on the mean vectors when sparsity is desired in model estimation, for example, $\sum_{k=1}^{K}\sum_{j=1}^{p}p\left(\left|\mu_{kj}\right|,\lambda_{3}\right)$, which can be used to identify variables that have no effect on clustering. Under the simulation settings presented in this article, preliminary numerical experiments show that this penalty does not exhibit a significant effect on network estimation.

Advancing from LVGGM [1], the proposed method can further deal with heterogeneity and outliers, and employs a non-convex regularization technique with well-established oracle properties. This method simultaneously achieves robust identification of heterogeneity patterns and estimation of a consensus network structure accounting for latent variables. The proposed objective function (2) can be simplified to mixture GGM with the same precision matrix by letting $\lambda_{2}=\infty$ and $\pi_{0}=0$. Compared to other robust clustering methods aimed at approximately Gaussian clusters, the inclusion of the constant mixture component $\pi_{0}\delta$ enables a smooth, mixture-type transition between clusters and outliers, and has desirable theoretical properties [34]. Note that points in low-density regions are identified as outliers even if they are generated by a Gaussian distribution. While this may mislabel some Gaussian samples as outliers, such samples generally have minimal impact on clustering outcomes. In practical applications, this conservative strategy helps improve the overall performance in handling data heterogeneity and outliers.

### 2.3 | Practical and Computational Considerations

Our algorithm is structured with two nested loops, which are indexed by superscript (m) for the outer loop and (m,t) for the t-th inner loop under m-th outer loop, respectively. The initial value of a parameter is denoted by the superscript (0). For the initial value $\mu_{\left(K\right)}^{\left(0\right)},\delta^{\left(0\right)},S^{\left(0\right)},L^{\left(0\right)}$, and $\pi^{\left(0\right)}$, we conduct hierarchical clustering utilizing the Ward method and cut the dendrogram at K+1 to obtain the initial clustering. This procedure is implemented with hclust() function. We initially designate the subgroup with the fewest samples as the outliers. Additionally, under each m-th outer loop, we initialize $S^{\left(m,0\right)}=S^{\left(0\right)}$ as the identity matrix and $L^{\left(m,0\right)}=L^{\left(0\right)}$ as the zero matrix.

In the outer loop, we employ the expectation-maximization (EM) algorithm to optimize the proposed objective function. Denote the latent cluster assignment matrix as $w\in R^{n\times (K+1)}$, where its (i,j)-th entry $w_{ik}=1$ indicates the sample i belongs to the subgroup k and $w_{ik}=0$ otherwise for k=0,…,K, and k=0 represents the outliers. Denote $𝓚=\left\{\mu_{\left(K\right)},\delta ,S,L,\pi \right\}$ as the set of all unknown parameters. The penalized objective function (2) for the complete data can be formulated as:

$$𝓛(𝓚|X,w)=\frac{1}{n}\sum_{i=1}^{n}\left(w_{i0}\left(\text{log}\pi_{0}+\text{log}\delta \right)\right)\;+\frac{1}{n}\sum_{i=1}^{n}\left(\sum_{k=1}^{K}w_{ik}\left(\text{log}\pi_{k}+\text{log}f_{k}\left(x_{i};\mu_{k},{\left(S-L\right)}^{-1}\right)\right)\right)-𝒫(S,L),$$

where $𝒫(S,L)=\sum_{i\neq j}p\left(\left|S_{ij}\right|;\lambda_{1}\right)+\sum_{j}p\left(\sigma_{j}(L);\lambda_{2}\right)$. In the m-th E-step of the EM algorithm, the conditional expectation of the complete data log-likelihood function is denoted as:

(3) $$𝒬\left(C;K^{\left(m-1\right)}\right)={𝔼}_{\gamma |X,K^{\left(m-1\right)}}[ℒ(K|X,\gamma )]\;=\frac{1}{n}\sum_{i=1}^{n}\left(\gamma_{i0}^{\left(m\right)}\left(\text{log}\pi_{0}+\text{log}\delta \right)\right)\;+\frac{1}{n}\sum_{i=1}^{n}\left(\sum_{k=1}^{K}\gamma_{ik}^{\left(m\right)}\left(\text{log}\pi_{k}+\text{log}f_{k}\left(x_{i};\mu_{k},{\left(S-L\right)}^{-1}\right)\right)\right)-𝒫(S,L),$$

where $\gamma_{ik}^{\left(m\right)}$ is the conditional expectation of the unobserved $w_{ik}$, which depends on the estimates obtained from the (m−1)-th step and is obtained by:

(4) $$\gamma_{ik}^{\left(m\right)}=\frac{\pi_{k}^{\left(m-1\right)}f_{k}\left(x_{i};\mu_{k}^{\left(m-1\right)},{\left(S^{\left(m-1\right)}-L^{\left(m-1\right)}\right)}^{-1}\right)}{\pi_{0}^{\left(m-1\right)}\delta^{\left(m-1\right)}+\sum_{k=1}^{K}\pi_{k}^{\left(m-1\right)}f_{k}\left(x_{i};\mu_{k}^{\left(m-1\right)},{\left(S^{\left(m-1\right)}-L^{\left(m-1\right)}\right)}^{-1}\right)},$$

for k=1,…,K, and $\gamma_{i0}^{\left(m\right)}=1-\sum_{k=1}^{K}\gamma_{ik}^{\left(m\right)}$. Following Coretto and Hennig [30], we apply the constraint $\frac{1}{n}\sum_{i=1}^{n}\gamma_{i0}\leq \pi_{\text{max}}$ during the update, where $0<\pi_{max}<1$ is a fixed threshold representing the maximum allowable proportion of outliers. We set $\pi_{\text{max}}=0.5$ to align with the standard robustness criteria.

In the M-step, maximizing (3) with respect to $\pi ,\mu_{\left(K\right)},\delta ,S,L$ yields the update of parameters. The update is given by:

(5) $$\pi_{k}^{\left(m\right)}=\frac{1}{n}\sum_{i=1}^{n}\gamma_{ik}^{\left(m\right)},k=1,\ldots ,K,\;\pi_{0}^{\left(m\right)}=1-\sum_{k=1}^{K}\pi_{k}^{\left(m\right)},\;\mu_{k}^{\left(m\right)}=\sum_{i=1}^{n}\gamma_{ik}^{\left(m\right)}x_{i},\;\delta^{\left(m\right)}=1/\prod_{j=1}^{p}2\sqrt{3\left(C_{2j}^{\left(m\right)}-{\left(C_{1j}^{\left(m\right)}\right)}^{2}\right)},$$

where

$$C_{1j}^{\left(m\right)}=\frac{1}{n}\sum_{i=1}^{n}\frac{\gamma_{i0}^{\left(m\right)}}{\pi_{0}^{\left(m\right)}}x_{ij},C_{2j}^{\left(m\right)}=\frac{1}{n}\sum_{i=1}^{n}\frac{\gamma_{i0}^{\left(m\right)}}{\pi_{0}^{\left(m\right)}}x_{ij}^{2}.$$

Indeed, δ is updated using the actual volume occupied by the outliers, which increases the ability to discriminate between outliers and others.

The update for **S, L** is equivalent to solving:

(6) $$\left\{S^{\left(m\right)},L^{\left(m\right)}\right\}=\underset{\left\{S,L\right\}}{argmin}\left(-logdet(S-L)+tr\left({\tilde{\Sigma }}^{\left(m\right)}(S-L)\right)+𝒫(S,L)\right),$$

where ${\tilde{\Sigma }}^{\left(m\right)}$ is the pseudo sample covariance matrix defined by:

(7) $${\tilde{\Sigma }}^{\left(m\right)}=\sum_{k=1}^{K}\sum_{i=1}^{n}\gamma_{ik}^{\left(m\right)}\left(x_{i}-\mu_{k}^{\left(m\right)}\right)\;{\left(x_{i}-\mu_{k}^{\left(m\right)}\right)}^{⊤}/n.$$

In the inner loop, we apply the alternating direction method of multipliers (ADMM) algorithm to optimize objective function (6), and obtain $\Theta^{\left(m\right)}=S^{\left(m\right)}-L^{\left(m\right)}$. The optimization is then transformed into the constrained objective function as follows:

$$minimize\;-logdet(S-L)+tr\left({\tilde{\Sigma }}^{\left(m\right)}(S-L)\right)+𝒫(S,L)\;\text{subject to}\;A=S-L,L≽0,A≽0.$$

Optimizing the constrained objective function is equivalent to optimizing the augmented Lagrangian function:

$$-log\;det(A)+tr\left({\tilde{\Sigma }}^{\left(m\right)}A\right)+𝒫(S,L)+tr(U(A-S+L))+\frac{\rho }{2}\|A-S+L{\|}_{F}^{2},$$

where the dual matrix $U\in {\mathbb{R}}^{p\times p}$ is the Lagrange multiplier, $\|\cdot {\|}_{F}$ denote the Frobenius norm, and ρ is the penalty parameter, usually fixed at 1 . We then compute $S^{\left(m\right)},L^{\left(m\right)}$ through iterative steps (a–d). For the t-th inner iteration, the updates for $S^{\left(m,t\right)},L^{\left(m,t\right)},U^{\left(m,t\right)}$, and $A^{\left(m,t\right)}$ are derived based on the results from the (m,t−1)-th inner iteration.

Step (a): Update $A^{\left(m,t\right)}$ by:

$$A^{\left(m,t\right)}=\text{arg}\underset{A>0}{\text{min}}(-\text{logdet}(A)+\text{tr}\left({\tilde{\Sigma }}^{\left(m\right)}A\right)\;+\text{tr}\left(U^{\left(m,t-1\right)}\left(A-S^{\left(m,t-1\right)}+L^{\left(m,t-1\right)})\right)+\frac{\rho }{2}{\left\|A-S^{\left(m,t-1\right)}+L^{\left(m,t-1\right)}\right\|}_{F}^{2}\right).$$

Following Witten and Tibshirani [35], the solution:

(8) $$A^{\left(m,t\right)}=Proj\left(V\tilde{D}V^{⊤},\epsilon \right),$$

where ${VDV}^{⊤}$ is the eigen decomposition of $\rho \left(S^{\left(m,t-1\right)}-L^{\left(m,t-1\right)}\right)-{\tilde{\Sigma }}^{\left(m\right)}-U^{\left(m,t-1\right)},\tilde{D}$ is a diagonal matrix with the j-th diagonal element $\frac{1}{2\rho }\left(D_{jj}+{\left(D_{jj}^{2}+4\rho \right)}^{1/2}\right)$, and $D_{jj}$ is the j-th diagonal element of D. Proj(⋅) is the projection operator. For any real symmetric matrix M, let $M=V_{M}diag(\sigma (M))V_{M}^{⊤}$ be its eigenvalue decomposition, define $Proj(M,\epsilon )=V_{M}diag(\tilde{\sigma }(M))V_{M}^{⊤}$, where $\tilde{\sigma }(M)={\left({\tilde{\sigma }}_{1}(M),\ldots ,{\tilde{\sigma }}_{p}(M)\right)}^{⊤}$ and ${\tilde{\sigma }}_{j}(M)=max\left\{\sigma_{j}(M),\epsilon \right\}$. ϵ≥0 is a small constant to ensure the positive definiteness or positive semi-definiteness of M. Here, ϵ is fixed at 0.001 in our simulation.

Step (b): Update $L^{\left(m,t\right)}$ by:

$$L^{\left(m,t\right)}=arg\;\underset{L≽0}{min}\left(tr\left(U^{\left(m,t-1\right)}L\right)+\frac{\rho }{2}{\left\|A^{\left(m,t\right)}-S^{\left(m,t-1\right)}+L\right\|}_{F}^{2}+\sum_{j}p\left(\sigma_{j}(L);\lambda_{2}\right)\right).$$

It can be solved that:

(9) $$L^{\left(m,t\right)}=Proj\left(P\tilde{Q}P^{⊤},0\right).$$

Denote $PQP^{⊤}$ be the eigen decomposition of $S^{\left(m,t-1\right)}-A^{\left(m,t\right)}$ $U^{\left(m,t-1\right)}/\rho ,\tilde{Q}$ is a diagonal matrix with the j-th diagonal element $ℋ\left(Q_{jj};\lambda_{2}/\rho \right)$, and $Q_{jj}$ is the j-th diagonal element of Q.ℋ(⋅;λ) is the proximal operator for MCP penalty with tuning parameter λ, defined as:

$$ℋ(c;\lambda )=\left\{\begin{array}{ll} \frac{ST_{\lambda }(c)}{\left(1-1/a\right)} & \text{if}\;|c|⩽a\lambda ,\\ c & \text{if}\;|c|>a\lambda , \end{array}\right.$$

where $ST_{\lambda }(c)=sign(c)\cdot max(|c|-\lambda ,0)$ is the soft thresholding operator, and *a* is taken as 3 which controls the concavity of the MCP penalty function.

Step (c): Update $S^{\left(m,t\right)}$ by:

$$S^{\left(m,t\right)}=arg\underset{S}{min}\left(-tr\left(U^{\left(m,t-1\right)}S\right)+\frac{\rho }{2}{\left\|A^{\left(m,t\right)}-S+L^{\left(m,t\right)}\right\|}_{F}^{2}+\sum_{i\neq j}p\left(\left|S_{ij}\right|;\lambda_{1}\right)\right).$$

We can get a closed-form solution of

(10) $$S^{\left(m,t\right)}=ℋ\left(A^{\left(m,t\right)}+L^{\left(m,t\right)}+U^{\left(m,t-1\right)}/\rho ;\lambda_{1}/\rho \right).$$

Step (d): Update $U^{\left(m,t\right)}$ by:

(11) $$U^{\left(m,t\right)}=U^{\left(m,t-1\right)}+\rho \left(A^{\left(m,t\right)}-S^{\left(m,t\right)}+L^{\left(m,t\right)}\right).$$

Denote the primal residual as $r^{\left(m,t\right)}={\left\|A^{\left(m,t\right)}-S^{\left(m,t\right)}+L^{\left(m,t\right)}\right\|}_{F}$. We stop the iteration when the residual is small enough such

**ALGORITHM 1:** 

**Initialize** $\mu_{\left(K\right)}^{\left(0\right)},\delta^{\left(0\right)},S^{\left(0\right)},L^{\left(0\right)}$, and $\pi^{\left(0\right)}$.
**Repeat for** *m* = 1, …, ***M*** as follows:
(1) E-step: Update the subgroup assignment $\gamma_{ik}^{\left(m\right)}$ using (4).
(2) M-step: Update $\mu_{\left(K\right)}^{\left(m\right)},\delta^{\left(m\right)},S^{\left(m\right)},L^{\left(m\right)},\pi^{\left(m\right)}$.
(2.1) Update $\mu_{\left(K\right)}^{\left(m\right)},\delta^{\left(m\right)},\pi^{\left(m\right)}$ using (5).
(2.2) Calculate ${\tilde{\Sigma }}^{\left(m\right)}$ by (7), and update $S^{\left(m\right)},L^{\left(m\right)}$ as the minimizer of (6).
**Repeat for** $t=1,\ldots ,T^{\left(m\right)}$ as follows:
(a) Update $A^{\left(m,t\right)}$ using (8).
(b) Update $L^{\left(m,t\right)}$ using (9).
(c) Update $S^{\left(m,t\right)}$ using (10).
(d) Update the dual variable $U^{\left(m,t\right)}$ using (11).
Repeat the steps (a–d) until ${\left\|r^{\left(m,t\right)}\right\|}_{F}<0.001$. Take $S^{\left(m\right)}=S^{\left(m,T^{\left(m\right)}\right)},L^{\left(m\right)}=L^{\left(m,T^{\left(m\right)}\right)}$.
Repeat the steps (1) and (2) until $\frac{{\left\|\mu_{\left(K\right)}^{\left(m\right)}-\mu_{\left(K\right)}^{\left(m-1\right)}\right\|}_{F}}{{\left\|\mu_{\left(K\right)}^{\left(m-1\right)}\right\|}_{F}}+\frac{{\left\|\Theta^{\left(m\right)}-\Theta^{\left(m-1\right)}\right\|}_{F}}{{\left\|\Theta^{\left(m-1\right)}\right\|}_{F}}<0.001$.

that ${\left\|r^{\left(m,t\right)}\right\|}_{F}<0.001$ in our numerical study. The final estimates obtained upon convergence of the inner loop are adopted as the updated $S^{\left(m\right)}$ and $L^{\left(m\right)}$ in the outer loop. Define $T^{\left(m\right)}$ as the terminating step of the inner loop. Upon completing $T^{\left(m\right)}$ iterations, we update the parameters as $S^{\left(m\right)}=S^{\left(m,T^{\left(m\right)}\right)}$ and $L^{\left(m\right)}=L^{\left(m,T^{\left(m\right)}\right)}$. Subsequently, we substitute $S^{\left(m-1\right)}$ and $L^{\left(m-1\right)}$ in Equation (4) with $S^{\left(m\right)}$ and $L^{\left(m\right)}$, respectively, and then repeat the homotopy process. Overall convergence is achieved when ${\left\|\mu_{\left(K\right)}^{\left(m\right)}-\mu_{\left(K\right)}^{\left(m-1\right)}\right\|}_{F}/\|\mu_{\left(K\right)}^{\left(m-1\right)}{\|}_{F}+\Theta^{\left(m\right)}-\Theta^{\left(m-1\right)}{\|}_{F}/\|\Theta^{\left(m-1\right)}{\|}_{F}<0.001$, and the terminating step of outer loop is denoted as M. We summarize the above descriptions in Algorithm 1.

Next, we consider the selection of tuning parameters ($\lambda_{1},\lambda_{2}$). When p is large, it becomes imperative to consider computational efficiency when exploring a grid of $\lambda_{1}\times \lambda_{2}$ penalty parameter combinations. Unless employing a coarse grid structure for these parameters or dedicating considerable time to pinpointing the optimal set, it is advisable to use information criteria rather than computationally intensive techniques like cross-validation. To mitigate computational burdens, we follow a line search approach for tuning parameter selection, as suggested in Danaher et al. [10]. In detail, we fix $\lambda_{2}$ at its median value of the given range and conduct a grid search over $\lambda_{1}$. With tuned $\lambda_{1}$, we conduct a grid search over $\lambda_{2}$. The tuning parameters are selected to minimize the following AIC-type selection criterion (with irrelevant items omitted):

(12) $$\text{AIC}\left(\lambda_{1},\lambda_{2}\right)=-\sum_{i=1}^{n}\text{log}\left({\hat{\pi }}_{0}\hat{\delta }+\sum_{k=1}^{K}{\hat{\pi }}_{k}f_{k}\left(x_{i};{\hat{\mu }}_{k},{\left(\hat{S}-\hat{L}\right)}^{-1}\right)\right)+2(p\hat{r}+\hat{s}),$$

where $\hat{s}$ is the number of non-zero elements in $\hat{S},\hat{r}$ is the rank of $\overset{ˆ}{L}$, respectively. The number of subgroup K can either be pre-determined based on domain-specific prior knowledge (e.g., known biological subtypes or clinical stratification criteria) or selected using cross-validation or information criteria [36–38].

*Remark* 1. Since the updates of both $L^{\left(m,t\right)}$ and $A^{\left(m,t\right)}$ involve the eigen decomposition of symmetric matrices, the computational complexity can be $O\left(p^{3}\right)$ when p is large. In practical applications, computation can be accelerated through several strategies. First, extract only the relevant eigencomponents to avoid a full decomposition by the Lanczos algorithm when updating $L^{\left(m,t\right)}$ and $A^{\left(m,t\right)}$ [39]. Second, the full eigen-decomposition can be replaced with a low-rank factorization (e.g., $L={ZZ}^{⊤},Z\in {\mathbb{R}}^{p\times r}$ ) with a pre-determined rank, which reduces the complexity to $O\left(pr^{2}\right)$ [40]. Third, leverage GPU and parallel computing to further enhance scalability for large-scale matrix processing [41].

*Remark* 2. If we consider distinct precision matrices with $\Theta_{k}=S_{k}-L_{k}$, the above algorithm can be modified by replacing the shared covariance matrix with subgroup-specific ${\tilde{\Sigma }}_{k}$, and then updating $S_{k}$ and $L_{k}$ for each subgroup via similar steps. In the case where $\Theta_{k}=S_{k}-L$ with a common low-rank component, L is first updated using a shared pseudo-covariance estimate $\tilde{\Sigma }$, after which subgroup-specific ${\tilde{\Sigma }}_{k}$ is estimated to update $S_{k}$ while keeping L fixed.

## 3 | Simulation Study

### 3.1 | Design

We conduct a series of simulations to test the performance of the proposed method in various scenarios. In our simulation settings, we fix the number of subgroups K=3, and the total sample size n=1500. The number of observed variables is set to p={40,100} and the rank of L matrix r={5,10}. For the mixture probabilities, $\pi =\left(\pi_{0},\pi_{1},\pi_{2},\pi_{3}\right),\pi_{0}=\{0\%,4\%,10\%\}$ is the expected proportion of noise component. We consider two settings for the other samples, namely balanced and imbalanced. Under the balanced setting, $\left(\pi_{1},\pi_{2},\pi_{3}\right)=\left(1-\pi_{0}\right)\times (1/3,1/3,1/3)$, and under the imbalanced setting $\left(\pi_{1},\pi_{2},\pi_{3}\right)=\left(1-\pi_{0}\right)\times (1/2,1/4,1/4)$. The normal observations are generated as follows. We first generate a sparse, symmetric, positive definite matrix $\bar{\Theta }\in {\mathbb{R}}^{\left(p+r)\times (p+r\right)}$, which can be decomposed into four submatrices.${\bar{\Theta }}_{1:p,1:p}$ corresponds to the interconnections among the observed variables and follows a random sparsity pattern with approximately 10% non-zero entries. ${\bar{\Theta }}_{(p+1):(p+r),1:p}$ reflects the interconnections between observed and latent variables, with a sparsity level of 70%.${\bar{\Theta }}_{\left(p+1):(p+r),(p+1):(p+r\right)}$ represents the interconnections among the latent ones, which is set to be a diagonal matrix indicating the independent latent variables. To obtain a positive definite matrix, we set $\tilde{\Theta }=\bar{\Theta }+\left(0.1+\left|\sigma_{\text{min}}(\bar{\Theta })\right|\right)I_{p+r}$, where $\sigma_{\text{min}}(\cdot )$ denotes the minimum eigenvalue of the matrix, and $I_{p+r}$ is the (p+r)×(p+r) identity matrix. The true values of parameter matrix can be calculated accordingly. $S≔{\tilde{\Theta }}_{1:p,1:p},L≔{\tilde{\Theta }}_{1:p,(p+1):(p+r)}{\left({\tilde{\Theta }}_{\left(p+1):(p+r),(p+1):(p+r\right)}\right)}^{-1}{\tilde{\Theta }}_{(p+1):(p+r),1:p}$, and Θ=S−L. There are $\tilde{p}=p/10$ variables having different mean values in different subgroups, while the other mean values are zero. Specifically, the first $\tilde{p}$ components of the mean vector $\mu_{k}$ are set to $\mu 1_{\tilde{p}}I(k=1)+\left(\mu 1_{\tilde{p}/2},-\mu 1_{\tilde{p}/2}\right)I(k=2)-\mu 1_{\tilde{p}}I(k=3)$, where $1_{\tilde{p}}$ is a vector of length $\tilde{p}$ with all components equal to 1 , and μ=1.5 unless stated otherwise. Subsequently, the samples in k-th subgroup are generated from the subgroup-specific distribution: $x_{i}∼𝒩\left(\mu_{k},\Theta^{-1}\right)$ for k=1,2,3. The outliers originate from a distribution obtained as the product of independent one-dimensional uniform distributions with support on the interval $[-20, 5{]}^{\tilde{p}/2}\times [-10, 10{]}^{\tilde{p}/2}$, and the remaining $p-\tilde{p}$ variables are generated from $𝒩\left(0,\Theta_{\tilde{p}:p,\tilde{p}:p}^{-1}\right)$.

We comprehensively evaluate the proposed method and compare it with the alternative methods. (a) LVGGM: Classic LVGGM in Chandrasekaran et al. [1], which is homogeneous model that does not consider the presence of outliers. (b) H-LVGGM: H-LVGGM incorporates only heterogeneity, without accounting for the noise components in (2). (c) OR-LVGGM: Employ LVGGM for each subgroup with true label separately. (d) Glasso: Graphical lasso without the consideration of both heterogeneity and outliers. (e) OR-Glasso: Employ Glasso for each subgroup with true label separately. (f) RH-GGM: GGM accounts for both heterogeneity and robustness while ignoring the latent variables. (g) H-GGM: Heterogeneity GGM based on mixture model with common sparse precision matrix. For fair comparison, all alternatives use MCP function for the sparsity of graph structure, except for the Glasso and OR-Glasso methods. The aforementioned methods can be broadly categorized into two categories: GGM-based and LVGGM-based. The GGM-based category encompasses Glasso, OR-Glasso, H-GGM, and RH-GGM, each using one tuning parameter for sparsity. The remaining methods fall into the LVGGM-based framework with two tuning parameters for both sparsity and low-rank. We employ AIC in all methods to determine the tuning parameters. Both the OR-Glasso and OR-LVGGM perform independent parameter selection for each subgroup. In our simulations, we fixed *K* to its true value for better comparability.

### 3.2 | Results

**FIGURE 2 |:**
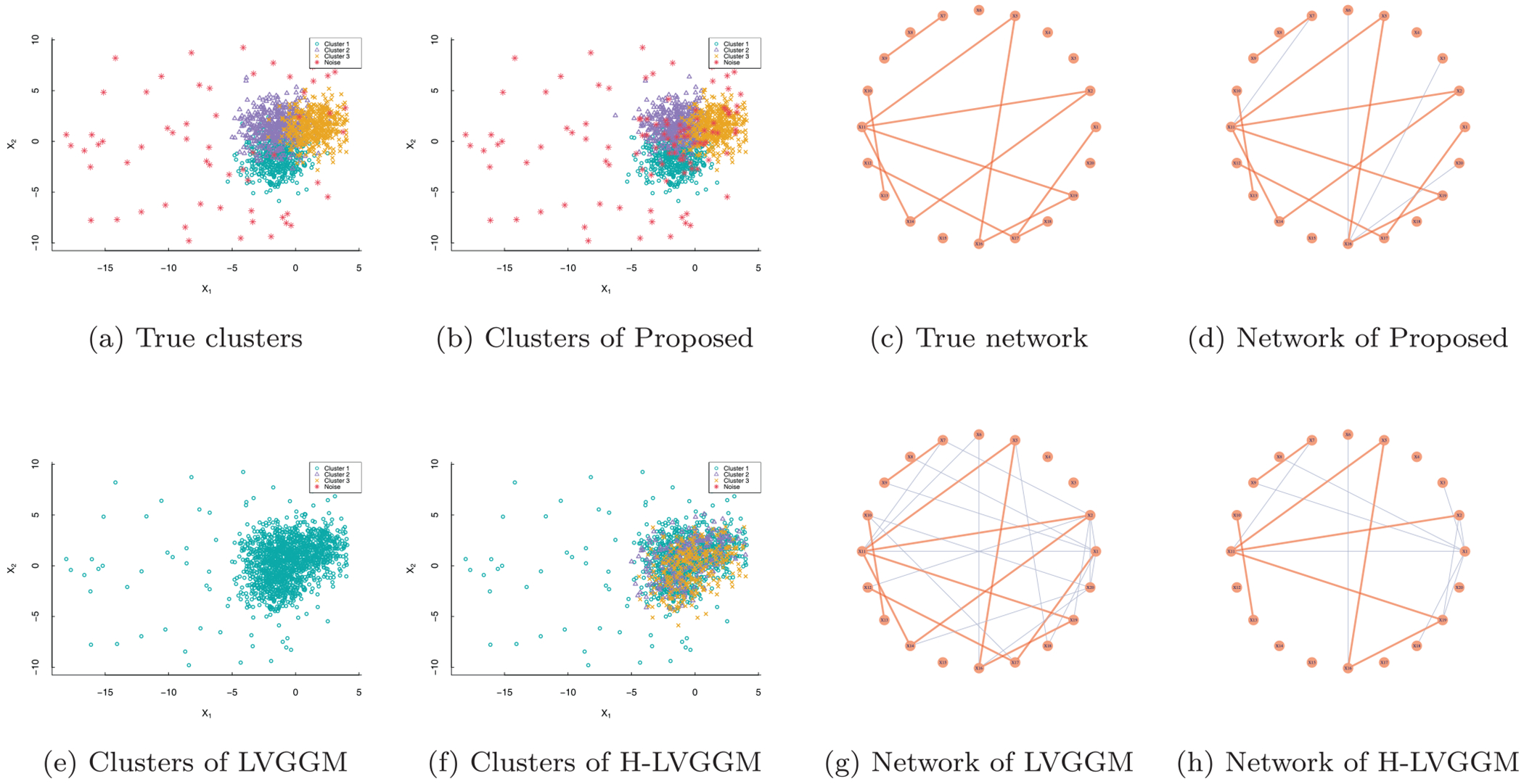
An example illustrating clustering results and network construction of the proposed method and some alternative methods.

To visually demonstrate the performance of the proposed method compared to some alternative methods, we use an example with 20 nodes and present the results in Figure 2. It shows that the proposed method outperforms the alternatives in network estimation, clustering, and outlier detection. When ignoring the heterogeneity, the resulting networks tend toward dense interconnections, as shown in panels (e) and (g). When outliers are not considered, the resulting clustering becomes disordered, which is obscured by outliers and further results in biased network estimates, as shown in panels (f) and (h). To quantitatively gauge the performance, we consider the following measures: (a) relative estimation errors of low-rank and sparse matrices in terms of Frobenius norm, denoted as $\|\overset{ˆ}{S}-S{\|}_{F}/\|S{\|}_{F},\|\hat{L}-L{\|}_{F}/L{\|}_{F}$ and $\|\overset{ˆ}{\Theta }-\Theta {\|}_{F}/\|\Theta {\|}_{F}$; (b) estimated rank $\hat{r}$; (c) true/false positive rates (TPR/FPR) for identifying non-zero off-diagonal elements of the matrix **S**; (d) ARI_*c*_ evaluates the accuracy of clustering using the adjusted Rand index (ARI) only for normal samples; (e) ARI_*o*_ evaluates the performance of outlier detection (measured by ARI based on correct identification of outliers); (f) ARI_*p*_ evaluates the accuracy of prediction using ARI computed on 500 independent test samples. Regarding the GGM-based methods, there is no estimate of L, so we set $\hat{S}=\hat{\Theta }$. For OR-Glasso and OR-LVGGM, all measures are averaged across all oracle subgroups.

With 100 replicates, the results under the balanced setting with *p* = 40 and *p* = 100 are summarized in Tables 1 and 2, respectively. The results for other settings are presented in Tables A1–A4 in Appendix A. The proposed approach is observed to have competitive performance across the whole spectrum of simulation. It achieves the lowest estimation errors among all competing methods in Table 1, and maintains this superior performance in the setting with *p* = 100. As a representative example, we consider Table 1, the setting of $r=5,\pi_{0}=0.04$. The proposed approach yields an estimation error of **Θ** at 0.143, significantly outperforming both LVGGM and H-LVGGM with mean errors of 0.273 and 0.258, respectively. This highlights the limitations of methods without accounting for heterogeneity and/or outliers. Although estimation accuracy gradually deteriorates for all methods as the outliers and rank increase, the proposed method can still preserve its relative advantage.

**FIGURE 3 |:**
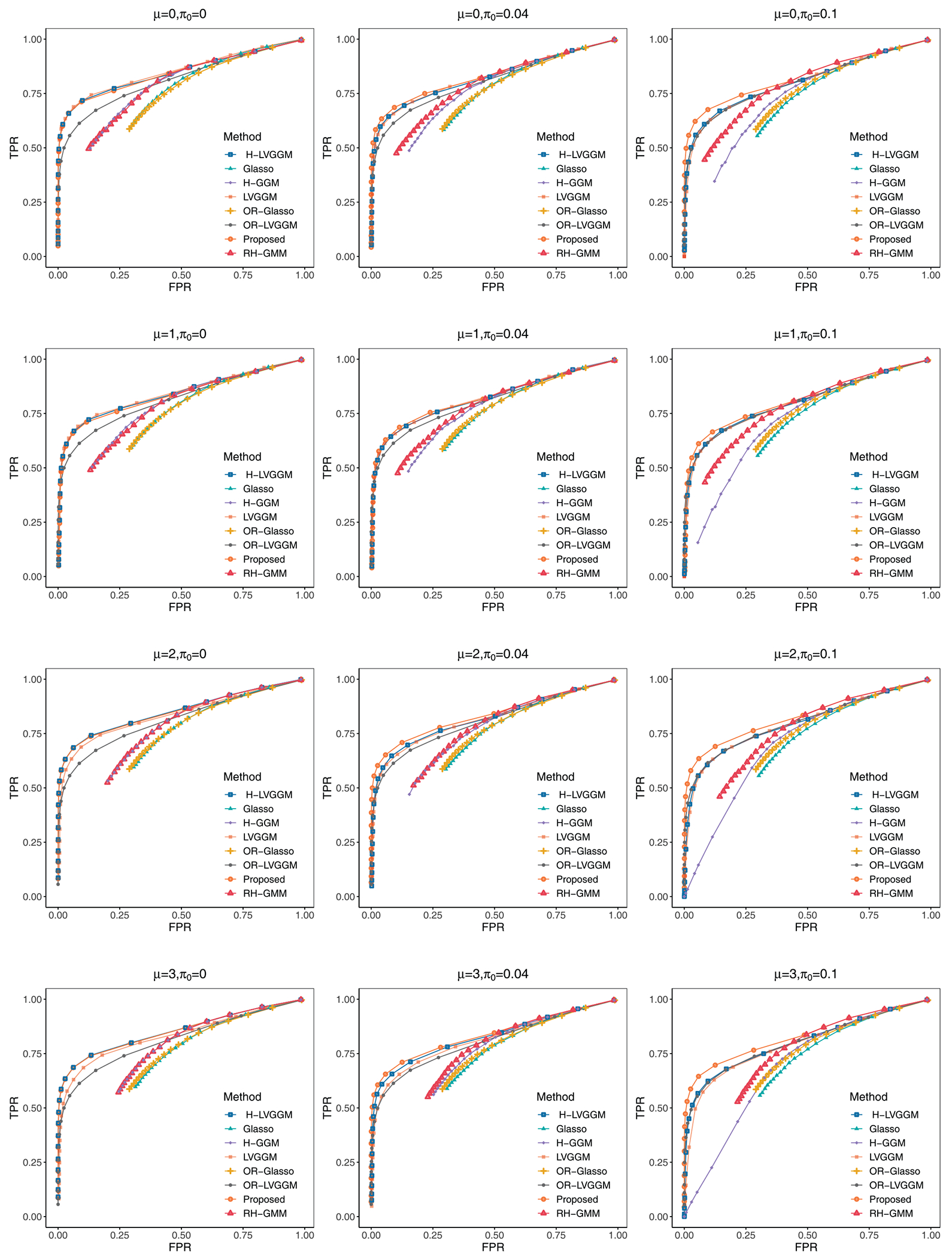
TPR-FPR curve across different combinations of *μ*, *π*_0_ under *p* = 40, *r* = 10 and fixed *λ*_2_ = 0.5.

**FIGURE A1 |:**
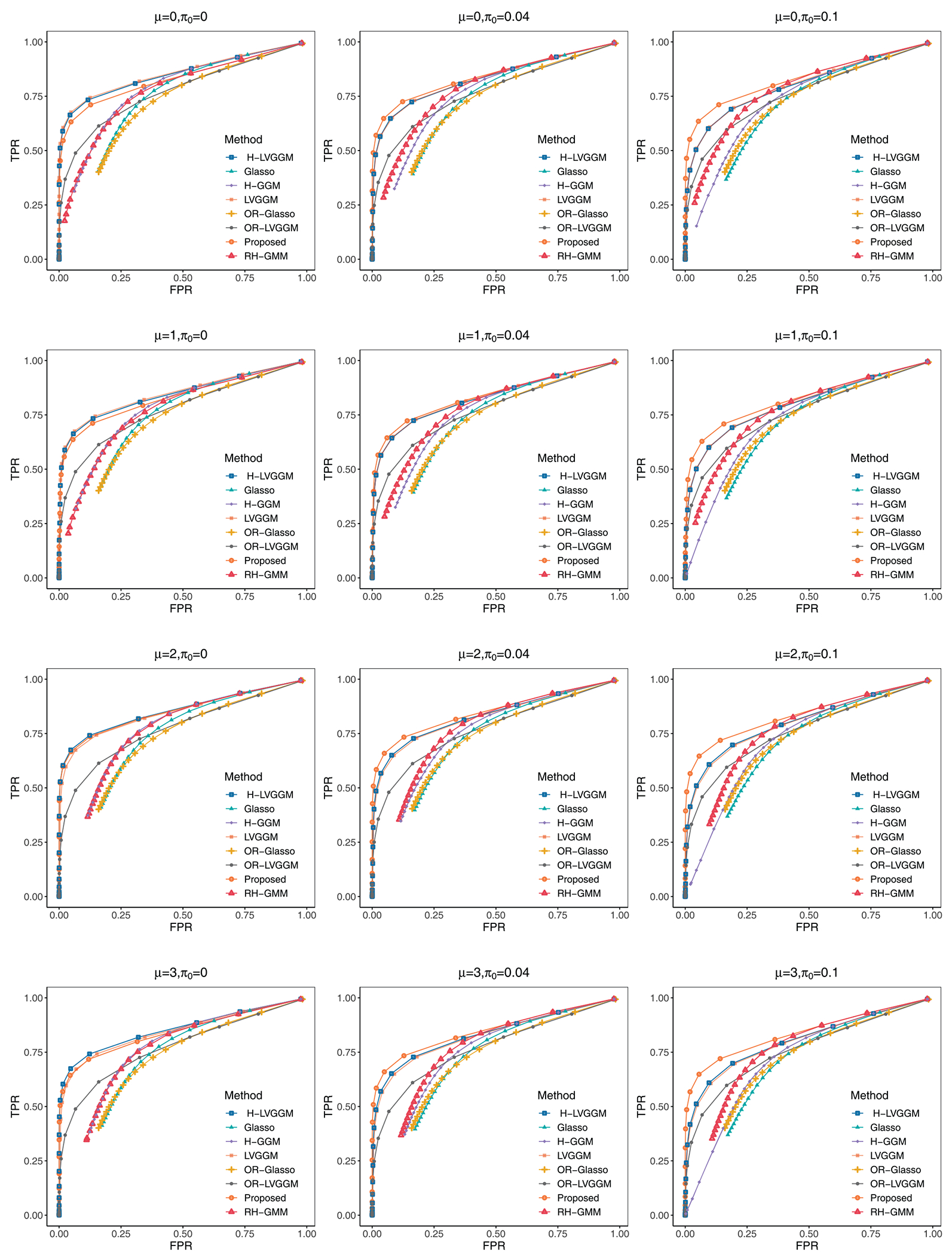
TPR-FPR curve across different combinations of $\mu ,\pi_{0}$ under p=40,r=10 and fixed $\lambda_{2}=0.5$.

As for the identification of network structure, we visually present the selection performance by plotting the λ_1_ -dependent variations in TPR and FPR with fixed λ_2_ = 0.5 under the balanced setting of *p* = 40, *r* = 10 in Figure 3. The corresponding results for *p* = 100, *r* = 10 are presented in Figure A1. Each curve is the average over 100 replicates. The closer the TPR-FPR curve approaches the upper-left corner, the better it is for identifying the true network structure. Under baseline conditions ($=0,\pi_{0}=0$) without heterogeneity and outliers, LVGGM and H-LVGGM demonstrate substantial performance overlap, as expected, and the proposed method also has competitive performance. Notably, LVGGM-based methods outperform GGM-based methods, indicating the significant advantages of incorporating latent variables for network estimation. Additionally, H-LVGGM and LVGGM have similar performance across most cases, as outliers cause cluster overlap in the H-LVGGM groupings, emphasizing the importance of addressing outliers. H-LVGGM has better performance when the distance between groups is large. As outliers increase, the superiority of the proposed method becomes evident, which confirms its effectiveness and robustness in handling heterogeneous datasets.

We further examine the effect of cluster separability by varying *μ*. The results are listed in Tables A5 and A6 in the Appendix A. It shows that all methods have improvement in clustering with increasing *μ*, that is, greater separability among subgroups, as expected. The proposed method still outperforms the alternative methods. When *μ* is sufficiently large, the impact of outliers is correspondingly weakened; the other methods achieve competitive clustering performance with the proposed, while remaining inferior in parameter estimates.

## 4 | Breast Cancer Data Analysis

Breast cancer is the most common type of cancer among women, encompassing a variety of molecular subtypes that exhibit distinct biological behaviors and clinical outcomes. Gene expression data has been collected and analyzed in quite a few studies. We analyze data collected in the Molecular Taxonomy of Breast Cancer International Consortium (METABRIC) study [42] and refer to the published literature [43] for information on sample and data collection and processing. The analysis includes 1904 samples that provide a comprehensive overview of gene expression profiles associated with breast cancer. We focus on 44 genes that overlap with the PAM50 [44], a well-established gene set used for breast cancer heterogeneity analysis.

**FIGURE 4 |:**
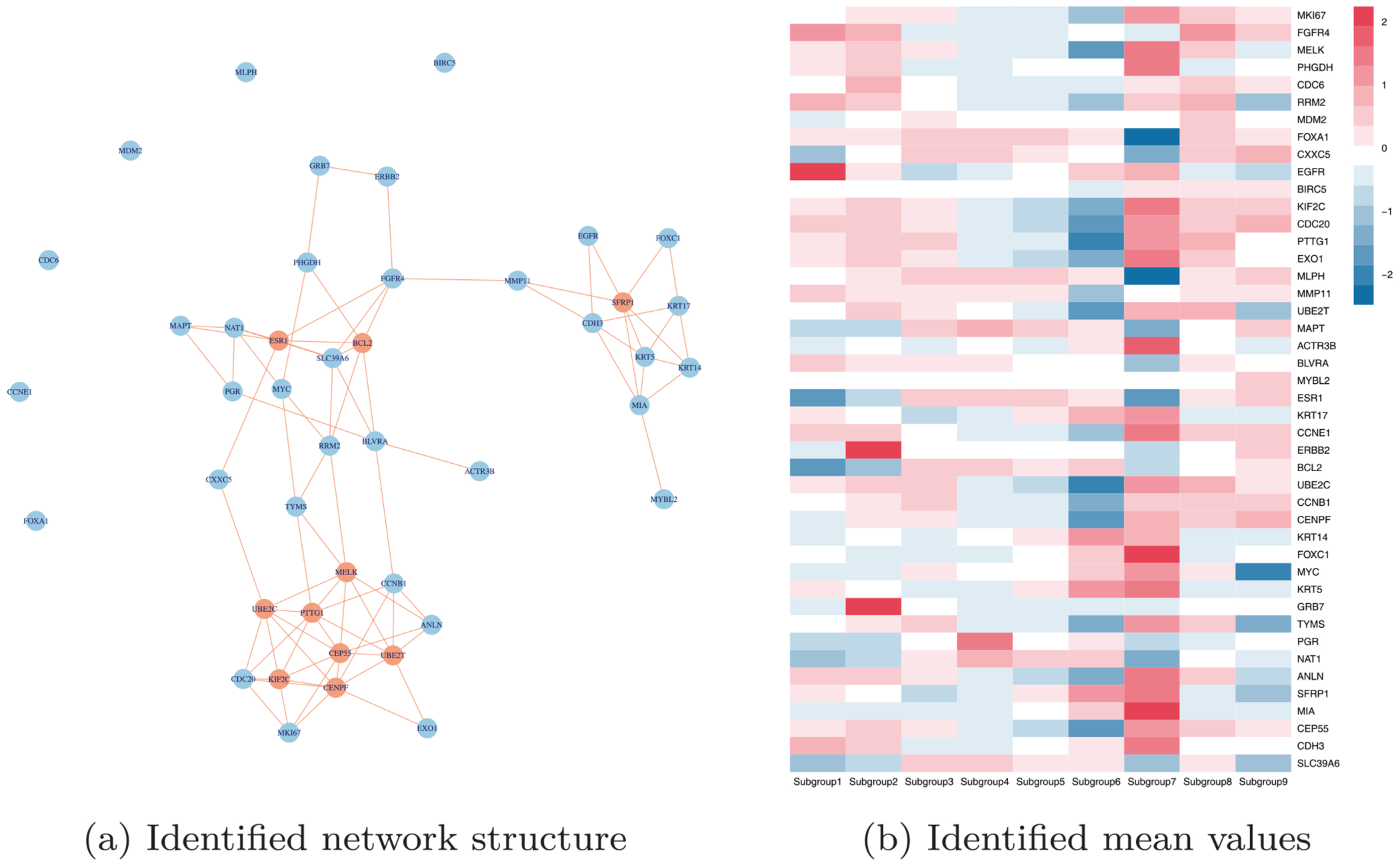
The identified heterogeneity and adjusted network estimation.

When implementing the proposed approach, we adopt AIC to determine the number of subgroups, with candidate integers ***K*** ≤ 10. Ultimately, a total of ***K*** = 9 subgroups are identified, containing 35, 211, 314, 326, 415, 113, 118, 142, and 56 samples, respectively. In addition, approximately 9.14% of the total observations are identified as outliers. The network estimate comprises 85 edges. Figure 4a visually displays the identified network structures. The top 10 genes with the highest connectivity in the network are marked in red. The identified hub genes, such as ESR1 (estrogen receptor), BCL2 (anti-apoptotic factor), MELK (mitotic kinase), and CEP55 (centrosomal protein), have been confirmed by multiple studies to be significantly associated with breast cancer proliferation, hormone response, and poor prognosis [45]. Additionally, the identification of novel hub genes such as UBE2T (ubiquitin-conjugating enzyme) and KIF2C (chromosome segregation regulator) further reveals the role of genomic instability in breast cancer progression. We also identify several significant interactions. For instance, the ESR1-BCL2 interaction edge suggests a synergistic regulatory mechanism between estrogen signaling pathways and apoptosis inhibition, which is closely linked to endocrine therapy resistance in breast cancer [46]. The interaction of ERBB2-GRB7 validates the characteristic receptor tyrosine kinase signaling pathway in HER2-positive subtypes, while the co-expression pattern of KRT5-KRT14-KRT17 reflects the keratin marker features of basal-like breast cancer [47].

**FIGURE 5 |:**
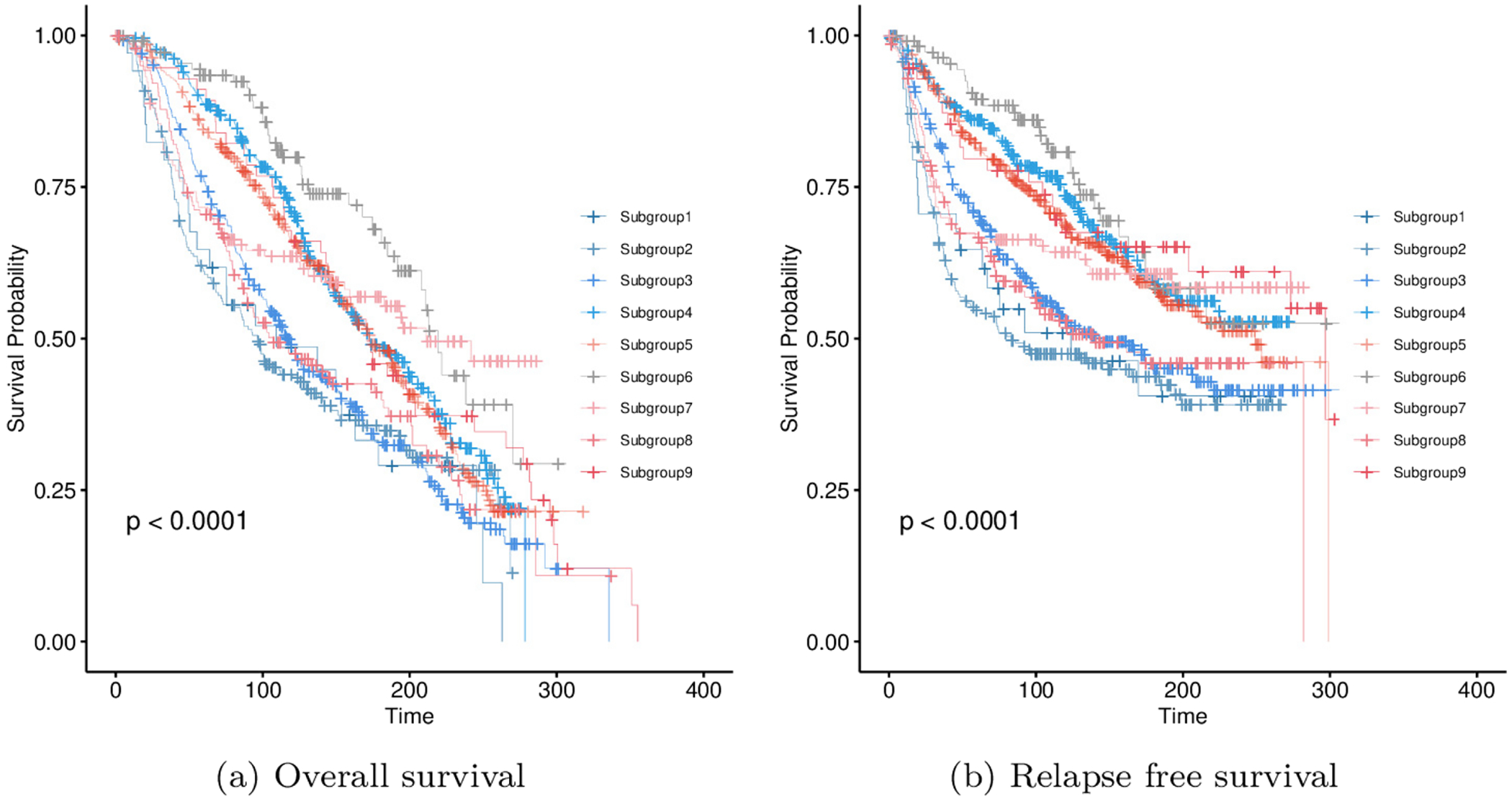
Comparison of survival time across different subgroups. Left: overall survival; Right: relapse free survival.

The proposed method also identifies the heterogeneity within breast cancer. Figure 4b shows the heatmap of the mean value of gene expression in each identified subgroup, implying significant differences. For example, the simultaneous high expression of GRB7 and ERBB2 is shown in the second subgroup. Such co-expression has been observed in specific clinical subtypes of breast cancer [48, 49]. In Figure 5, we compare overall survival and relapse-free survival time across the identified subgroups. Significant differences are observed (with *p* value < 0.0001), suggesting that the nine patient subgroups have notable clinical differences. We further compare the identified nine subgroups with two well-validated breast cancer classification systems, CLAUDIN and INTCLUST, which capture distinct biological dimensions in Tables A7 and A8 in Appendix B. The Rand index between the identified subgroups with these two subtyping are 0.768 and 0.821, respectively, outperforming H-LVGGM (0.719 and 0.797), suggesting certain consistency. Specifically, Claudin-low cases are distributed across Subgroup 1 to Subgroup 8 (with none observed in Subgroup 9), a pattern that closely reflects the core concept of the CLAUDIN subtype system that Claudin-low breast cancers are not confined to triple-negative (ER ^−^/HER2 ^−^) phenotypes but also include a substantial subset with ER positivity or HER2 positivity. This distribution reflects the well-documented intrinsic heterogeneity of the Claudin-low molecular cluster, as previously reported in genomic and clinical studies [50]. Subgroup 2 specifically maps to INTCLUST 5 and the HER2 subtype, consistent with the established finding that HER2-positive breast tumors are mainly concentrated in INTCLUST 5 in genomic classifications. Additionally, Subgroups 3, 4, and 5 primarily map to luminal subtypes: Subgroup 3 is dominated by Luminal B, while Subgroups 4 and 5 are dominated by Luminal A. Consistent with the genomic heterogeneity of luminal phenotypes, the LumB/LumA subtypes are distributed across INTCLUST 1, 2, 3, 6, 7, 8, and 9 [51]. Subgroup 7 exhibits high enrichment for Basal-like and Claudin-low subtypes according to CLAUDIN subtyping, while largely mapping to INTCLUST10 under the INTCLUST genomic classification. This agrees with the well-established association between Basal-like/Claudin-low phenotypes and INTCLUST10, a genomically defined cluster dominated by basal-like [52].

**FIGURE A2 |:**
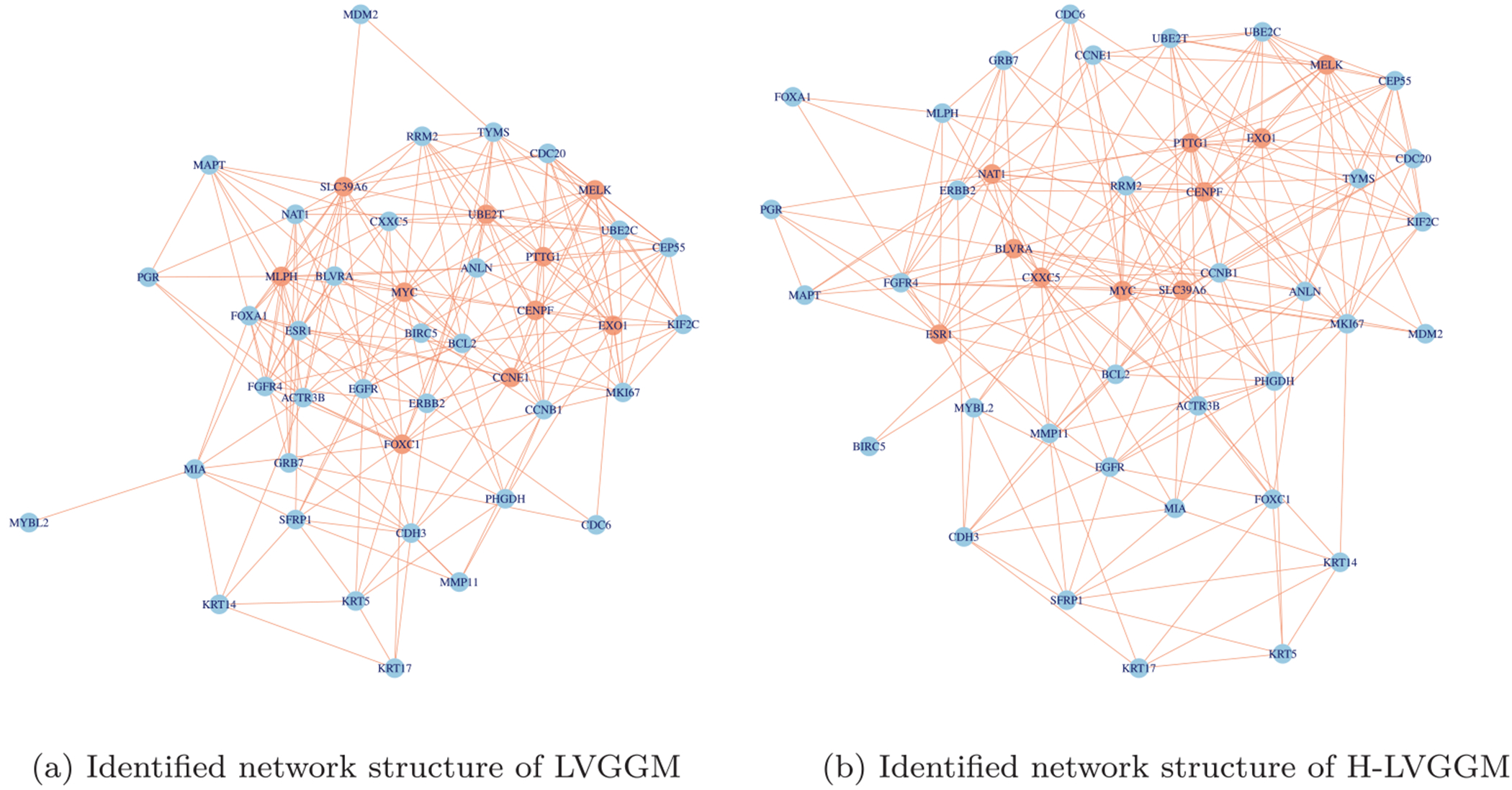
The network estimation of LVGGM and H-LVGGM.

Data is also analyzed using the alternative approaches. With each method, the number of identified subgroups ( ***K*** ), the number of edges (Num), and the sample negative log-likelihood (N-Loglik) are summarized in Table 3. The proposed method has the smallest sample negative log-likelihood, which indicates that it effectively captures the underlying structure of the data and provides a more accurate representation of the relationships among variables. In Figure A2 of Appendix B, we show the estimated network structures obtained from LVGGM and H-LVGGM. Both networks exhibit high connectivity density, which often corresponds to a high false discovery rate. In contrast, after adjusting for heterogeneity and outliers, the proposed method leads to a more lucid and more interpretable network.

## 5 | Discussion

This study introduces a flexible framework for latent variable Gaussian graphical modeling in the presence of heterogeneity and outliers. The proposed method yields a more lucid and direct network structure and can identify data heterogeneity and outliers simultaneously, which are equally important. The proposed method includes multiple existing ones as special cases and can advance the reliability of network estimation in complex real-world scenarios. The empirical validation on both synthetic and breast cancer data underscores its practical utility in uncovering biologically meaningful interactions.

There are several potential directions for future research. First, determining the number of clusters ***K*** remains a crucial challenge in practice. While we rely on information criteria for ***K*** selection, this approach can be unstable in high-dimensional or imbalanced data scenarios. In addition to using information criteria, ***K*** could be selected exploratorily by monitoring changes in the pseudo-likelihood across different values of ***K*** , similar to the approach used in TCLUST [53]. A detailed investigation of such selection strategies, however, is beyond the scope of the present study. Second, the Gaussianity assumption, though standard in graphical modeling, limits applicability to non-Gaussian data types common in biomedicine, such as count-based RNA-seq read counts or binary single-nucleotide polymorphism (SNP) data. Extending the framework to accommodate exponential family distributions or non-parametric assumptions (e.g., copula-based models [54]) would broaden its utility, though this would introduce computational complexities in estimating latent variables and mixture components. Third, integrating prior biological knowledge, such as curated gene regulatory networks or pathway databases, could refine network estimates. Incorporating priors or constrained optimization (to enforce known interactions) might reduce false discoveries, particularly in small-sample settings where data-driven estimation is unstable. Finally, beyond genomic applications, the framework’s robustness to heterogeneity, outliers, and latent confounding endows it with broad utility across disciplines: it can refine molecular interaction networks across other omics modalities, explore brain connectivity patterns in neuropsychiatric research, and disentangle context-dependent variations (e.g., seasonal shifts) in ecological data while accounting for extreme events.

## Supplementary Material

Supporting Information **Data S1:** sim70571-sup-0001-Supinfo.zip.

Additional supporting information can be found online in the Supporting Information section.

## Figures and Tables

**TABLE 1 |:** Simulation results of *p* = 40 under balanced setting: mean (standard deviation).

	Method	$\|\hat{S}-S{\|}_{F}/\|S{\|}_{F}$	$\|\hat{L}-L{\|}_{F}/\|L{\|}_{F}$	$\|\overset{ˆ}{\Theta }-\Theta {\|}_{F}/\|\Theta {\|}_{F}$	Rank	TPR	FPR	ARI_*c*_	ARI_*o*_	ARI_*p*_
*r* = 5, *π*_0_ = 0.04	Proposed	0.134 (0.058)	0.417 (0.146)	0.143 (0.056)	4.810 (2.282)	0.715 (0.106)	0.063 (0.113)	0.601 (0.424)	0.503 (0.055)	0.545 (0.362)
	LVGGM	0.270 (0.014)	0.496 (0.057)	0.273 (0.014)	5.040 (1.524)	0.644 (0.103)	0.056 (0.057)	—	—	—
	H-LVGGM	0.249 (0.016)	0.550 (0.118)	0.258 (0.016)	4.680 (2.849)	0.716 (0.091)	0.092 (0.065)	0.066 (0.158)	—	0.073 (0.148)
	OR-LVGGM	0.225 (0.022)	0.802 (0.138)	0.221 (0.018)	5.763 (1.274)	0.706 (0.069)	0.194 (0.036)	—	—	0.825 (0.028)
	Glasso	0.335 (0.013)	—	0.274 (0.008)	—	0.941 (0.043)	0.778 (0.047)	—	—	—
	OR-Glasso	0.191 (0.007)	—	0.191 (0.007)	—	0.887 (0.048)	0.630 (0.043)	—	—	0.830 (0.025)
	RH-GGM	0.274 (0.035)	—	0.208 (0.052)	—	0.849 (0.069)	0.457 (0.071)	0.526 (0.445)	0.502 (0.056)	0.475 (0.375)
	H-GGM	0.326 (0.018)	—	0.285 (0.015)	—	0.831 (0.077)	0.436 (0.095)	0.039 (0.124)	—	0.047 (0.117)
*r* = 10, *π*_0_ = 0.04	Proposed	0.158 (0.053)	0.513 (0.111)	0.171 (0.053)	6.670 (2.202)	0.659 (0.099)	0.078 (0.068)	0.504 (0.441)	0.473 (0.066)	0.458 (0.377)
	LVGGM	0.285 (0.011)	0.573 (0.051)	0.288 (0.010)	7.250 (1.708)	0.530 (0.100)	0.027 (0.017)	—	—	—
	H-LVGGM	0.259 (0.017)	0.598 (0.076)	0.264 (0.012)	6.660 (2.567)	0.659 (0.109)	0.110 (0.080)	0.050 (0.133)	—	0.060 (0.126)
	OR-LVGGM	0.250 (0.022)	0.840 (0.096)	0.236 (0.017)	7.307 (1.568)	0.642 (0.075)	0.191 (0.044)	—	—	0.812 (0.034)
	Glasso	0.355 (0.012)	—	0.274 (0.008)	—	0.950 (0.036)	0.827 (0.037)	—	—	—
	OR-Glasso	0.200 (0.006)	—	0.200 (0.006)	—	0.888 (0.039)	0.682 (0.041)	—	—	0.818 (0.032)
	RH-GGM	0.301 (0.029)	—	0.220 (0.048)	—	0.842 (0.062)	0.524 (0.082)	0.470 (0.444)	0.475 (0.062)	0.423 (0.373)
	H-GGM	0.346 (0.017)	—	0.286 (0.015)	—	0.827 (0.068)	0.508 (0.084)	0.038 (0.113)	—	0.046 (0.107)
*r* = 5, *π*_0_ = 0.1	Proposed	0.167 (0.051)	0.462 (0.112)	0.178 (0.053)	4.550 (1.598)	0.700 (0.100)	0.060 (0.039)	0.373 (0.347)	0.434 (0.047)	0.374 (0.295)
	LVGGM	0.312 (0.011)	0.562 (0.054)	0.316 (0.010)	5.080 (1.773)	0.559 (0.095)	0.040 (0.022)		—	—
	H-LVGGM	0.281 (0.008)	0.576 (0.055)	0.292 (0.009)	3.960 (1.197)	0.701 (0.094)	0.099 (0.033)	0.003 (0.004)	—	0.044 (0.009)
	OR-LVGGM	0.237 (0.022)	0.839 (0.139)	0.233 (0.018)	5.860 (1.226)	0.704 (0.065)	0.199 (0.035)	—	—	0.745 (0.031)
	Glasso	0.360 (0.012)	—	0.310 (0.008)	—	0.938 (0.039)	0.773 (0.051)	—	—	—
	OR-Glasso	0.196 (0.007)	—	0.196 (0.007)	—	0.886 (0.041)	0.628 (0.049)	—	—	0.748 (0.033)
	RH-GGM	0.299 (0.036)	—	0.249 (0.051)	—	0.839 (0.057)	0.447 (0.085)	0.334 (0.349)	0.437 (0.049)	0.332 (0.290)
	H-GGM	0.346 (0.015)	—	0.310 (0.011)	—	0.802 (0.070)	0.432 (0.059)	0.002 (0.003)	—	0.045 (0.009)
*r* = 10, *π*_0_ = 0.1	Proposed	0.205 (0.050)	0.603 (0.126)	0.220 (0.045)	5.880 (2.133)	0.644 (0.110)	0.107 (0.092)	0.330 (0.426)	0.402 (0.051)	0.263 (0.272)
	LVGGM	0.318 (0.009)	0.621 (0.050)	0.323 (0.008)	7.340 (2.185)	0.490 (0.090)	0.037 (0.019)	—	—	—
	H-LVGGM	0.289 (0.009)	0.628 (0.051)	0.298 (0.008)	6.220 (2.013)	0.643 (0.101)	0.119 (0.055)	0.002 (0.003)	—	0.045 (0.011)
	OR-LVGGM	0.265 (0.027)	0.901 (0.120)	0.253 (0.019)	7.490 (1.650)	0.631 (0.071)	0.191 (0.047)	—	—	0.721 (0.034)
	Glasso	0.376 (0.011)	—	0.309 (0.007)	—	0.941 (0.043)	0.815 (0.043)	—	—	—
	OR-Glasso	0.205 (0.007)	—	0.205 (0.007)	—	0.885 (0.038)	0.674 (0.045)	—	—	0.728 (0.034)
	RH-GGM	0.323 (0.029)	—	0.268 (0.045)	—	0.835 (0.065)	0.503 (0.088)	0.316 (0.418)	0.403 (0.049)	0.248 (0.260)
	H-GGM	0.362 (0.013)	—	0.312 (0.008)	—	0.801 (0.067)	0.484 (0.061)	0.002 (0.004)	—	0.047 (0.012)

**TABLE 2 |:** Simulation results of *p* = 100 under balanced setting: mean (standard deviation).

	Method	$\|\hat{S}-S{\|}_{F}/\|S{\|}_{F}$	$\|\hat{L}-L{\|}_{F}/\|L{\|}_{F}$	$\|\hat{\Theta }-\Theta {\|}_{F}/\|\Theta {\|}_{F}$	Rank	TPR	FPR	ARI*_c_*	ARI*_o_*	ARI*_p_*
*r* = 5, *π*_0_ = 0.04	Proposed	0.111 (0.015)	0.370 (0.051)	0.116 (0.015)	5.600 (0.778)	0.687 (0.049)	0.045 (0.020)	0.872 (0.162)	0.541 (0.056)	0.854 (0.150)
	LVGGM	0.280 (0.008)	0.534 (0.044)	0.282 (0.008)	6.057 (0.807)	0.480 (0.047)	0.019 (0.004)	—	—	—
	H-LVGGM	0.239 (0.012)	0.505 (0.060)	0.243 (0.013)	6.124 (1.423)	0.662 (0.060)	0.075 (0.032)	0.387 (0.236)	—	0.370 (0.223)
	OR-LVGGM	0.377 (0.059)	1.940 (0.510)	0.422 (0.065)	11.966 (2.226)	0.649 (0.045)	0.229 (0.029)	—	—	0.927 (0.022)
	Glasso	0.303 (0.008)	—	0.274 (0.007)	—	0.870 (0.031)	0.543 (0.045)	—	—	—
	OR-Glasso	0.226 (0.006)	—	0.226 (0.006)	—	0.749 (0.037)	0.376 (0.031)	—	—	0.936 (0.016)
	RH-GGM	0.214 (0.013)	—	0.177 (0.010)	—	0.753 (0.042)	0.273 (0.032)	0.877 (0.156)	0.543 (0.055)	0.842 (0.146)
	H-GGM	0.289 (0.011)	—	0.269 (0.012)	—	0.729 (0.043)	0.280 (0.039)	0.331 (0.241)	—	0.314 (0.227)
*r* = 10, *π*_0_ = 0.04	Proposed	0.120 (0.014)	0.441 (0.044)	0.131 (0.014)	10.730 (1.221)	0.631 (0.060)	0.040 (0.023)	0.867 (0.180)	0.542 (0.051)	0.845 (0.169)
	LVGGM	0.282 (0.009)	0.585 (0.035)	0.287 (0.008)	9.731 (1.429)	0.432 (0.040)	0.018 (0.004)	—	—	—
	H-LVGGM	0.241 (0.013)	0.543 (0.038)	0.247 (0.014)	10.692 (1.790)	0.629 (0.051)	0.078 (0.023)	0.343 (0.247)	—	0.330 (0.229)
	OR-LVGGM	0.339 (0.057)	1.523 (0.328)	0.389 (0.063)	15.962 (3.296)	0.583 (0.060)	0.188 (0.053)	—	—	0.927 (0.018)
	Glasso	0.319 (0.009)	—	0.275 (0.007)	—	0.884 (0.027)	0.621 (0.035)	—	—	—
	OR-Glasso	0.236 (0.004)	—	0.236 (0.004)	—	0.766 (0.031)	0.441 (0.033)	—	—	0.935 (0.015)
	RH-GGM	0.240 (0.013)	—	0.190 (0.011)	—	0.768 (0.036)	0.331 (0.030)	0.862 (0.183)	0.542 (0.051)	0.825 (0.170)
	H-GGM	0.307 (0.011)	—	0.276 (0.012)	—	0.738 (0.043)	0.332 (0.046)	0.321 (0.251)	—	0.307 (0.234)
*r* = 5, *π*_0_ = 0.1	Proposed	0.122 (0.013)	0.400 (0.058)	0.127 (0.013)	5.720 (0.866)	0.678 (0.053)	0.058 (0.028)	0.782 (0.132)	0.518 (0.045)	0.718 (0.115)
	LVGGM	0.321 (0.006)	0.584 (0.048)	0.324 (0.006)	6.050 (1.258)	0.440 (0.045)	0.034 (0.008)	—	—	—
	H-LVGGM	0.293 (0.007)	0.596 (0.045)	0.298 (0.007)	5.450 (1.140)	0.630 (0.067)	0.099 (0.031)	0.026 (0.108)	—	0.069 (0.091)
	OR-LVGGM	0.383 (0.032)	2.169 (0.288)	0.446 (0.029)	13.533 (1.550)	0.625 (0.033)	0.213 (0.020)	—	—	0.843 (0.018)
	Glasso	0.339 (0.006)	—	0.316 (0.005)	—	0.852 (0.031)	0.546 (0.042)	—	—	—
	OR-Glasso	0.229 (0.004)	—	0.229 (0.004)	—	0.745 (0.031)	0.378 (0.030)	—	1.000 (0.000)	0.849 (0.018)
	RH-GGM	0.224 (0.016)	—	0.192 (0.013)	—	0.749 (0.039)	0.273 (0.036)	0.770 (0.144)	0.517 (0.044)	0.679 (0.123)
	H-GGM	0.331 (0.007)	—	0.318 (0.006)	—	0.672 (0.052)	0.266 (0.030)	0.020 (0.095)	—	0.065 (0.081)
*r* = 10, *π*_0_ = 0.1	Proposed	0.131 (0.017)	0.470 (0.047)	0.143 (0.017)	10.850 (1.641)	0.623 (0.066)	0.052 (0.030)	0.749 (0.180)	0.503 (0.045)	0.681 (0.155)
	LVGGM	0.321 (0.006)	0.629 (0.035)	0.327 (0.006)	9.900 (2.380)	0.413 (0.054)	0.035 (0.010)	—	—	—
	H-LVGGM	0.294 (0.006)	0.620 (0.035)	0.302 (0.006)	9.930 (2.152)	0.602 (0.058)	0.107 (0.032)	0.045 (0.146)	—	0.085 (0.126)
	OR-LVGGM	0.379 (0.044)	1.736 (0.291)	0.438 (0.051)	15.527 (2.396)	0.581 (0.041)	0.201 (0.034)	—	—	0.832 (0.026)
	Glasso	0.352 (0.007)	—	0.317 (0.004)	—	0.864 (0.029)	0.615 (0.042)	—	—	—
	OR-Glasso	0.240 (0.004)	—	0.240 (0.004)	—	0.757 (0.031)	0.434 (0.032)	—	—	0.842 (0.023)
	RH-GGM	0.250 (0.015)	—	0.207 (0.016)	—	0.749 (0.038)	0.321 (0.038)	0.744 (0.206)	0.501 (0.044)	0.646 (0.173)
	H-GGM	0.343 (0.008)	—	0.323 (0.006)	—	0.690 (0.041)	0.313 (0.035)	0.038 (0.137)	—	0.080 (0.119)

**TABLE 3 |:** Comparison of alternative methods in breast cancer data analysis.

Method	Num	*K*	N-Loglik
Proposed	85	9	75441.73
LVGGM	217	—	83609.22
H-LVGGM	211	10	76893.44
Glasso	633	—	84560.83
RH-GGM	379	9	75612.13
H-GGM	365	10	77016.11

## Data Availability

The data that support the findings of this study are openly available at https://www.cbioportal.org/study/summary?id=brca_metabric. The R code for the proposed algorithm is publicly accessible at https://github.com/Linxi-Li/RH-LVGGM.

## Appendix A Additional Simulation Results

**TABLE A1 |:** Simulation results of *π*_0_ = 0% (balanced setting): mean (standard deviation).

	Method	$\|\hat{S}-S{\|}_{F}/\|S{\|}_{F}$	$\|\hat{L}-L{\|}_{F}/\|L{\|}_{F}$	$\|\overset{ˆ}{\Theta }-\Theta {\|}_{F}/\|\Theta {\|}_{F}$	Rank	TPR	FPR	ARI*_c_*	ARI_*p*_
*p* = 40, *r* = 5	Proposed	0.098 (0.045)	0.349 (0.120)	0.106 (0.044)	5.220 (2.329)	0.731 (0.083)	0.039 (0.049)	0.772 (0.310)	0.763 (0.309)
	LVGGM	0.197 (0.009)	0.418 (0.068)	0.198 (0.008)	4.510 (0.870)	0.795 (0.081)	0.126 (0.057)	—	—
	H-LVGGM	0.118 (0.057)	0.382 (0.145)	0.125 (0.056)	4.830 (1.832)	0.745 (0.095)	0.061 (0.086)	0.640 (0.416)	0.634 (0.414)
	OR-LVGGM	0.219 (0.026)	0.774 (0.140)	0.213 (0.020)	5.750 (1.265)	0.718 (0.059)	0.192 (0.034)	—	0.882 (0.026)
	Glasso	0.292 (0.013)	—	0.214 (0.007)	—	0.953 (0.035)	0.777 (0.045)	—	—
	OR-Glasso	0.189 (0.007)	—	0.189 (0.007)	—	0.895 (0.041)	0.637 (0.045)	—	0.886 (0.026)
	RH-GGM	0.250 (0.026)	—	0.163 (0.034)	—	0.867 (0.059)	0.491 (0.092)	0.768 (0.310)	0.762 (0.308)
	H-GGM	0.260 (0.027)	—	0.180 (0.044)	—	0.865 (0.072)	0.498 (0.113)	0.598 (0.423)	0.596 (0.423)
*p* = 40, *r* = 10	Proposed	0.131 (0.050)	0.460 (0.109)	0.135 (0.047)	7.080 (2.321)	0.697 (0.105)	0.081 (0.105)	0.701 (0.355)	0.700 (0.359)
	LVGGM	0.219 (0.014)	0.516 (0.059)	0.217 (0.014)	7.250 (2.096)	0.655 (0.132)	0.084 (0.072)	—	—
	H-LVGGM	0.166 (0.055)	0.516 (0.131)	0.167 (0.054)	7.100 (3.154)	0.692 (0.128)	0.097 (0.104)	0.457 (0.439)	0.459 (0.444)
	OR-LVGGM	0.248 (0.025)	0.831 (0.101)	0.231 (0.017)	7.057 (1.566)	0.657 (0.067)	0.198 (0.042)	—	0.866 (0.032)
	Glasso	0.319 (0.014)	—	0.217 (0.007)	—	0.958 (0.030)	0.837 (0.050)	—	—
	OR-Glasso	0.197 (0.007)	—	0.197 (0.007)	—	0.896 (0.034)	0.685 (0.047)	—	0.876 (0.030)
	RH-GGM	0.354 (0.338)	—	0.264 (0.415)	—	0.831 (0.180)	0.520 (0.138)	0.650 (0.378)	0.648 (0.379)
	H-GGM	0.299 (0.029)	—	0.209 (0.049)	—	0.868 (0.066)	0.539 (0.107)	0.398 (0.438)	0.399 (0.440)
*p* = 100, *r* = 5	Proposed	0.108 (0.015)	0.369 (0.075)	0.112 (0.014)	5.440 (0.914)	0.696 (0.050)	0.047 (0.044)	0.915 (0.145)	0.901 (0.153)
	LVGGM	0.209 (0.006)	0.480 (0.049)	0.213 (0.005)	5.850 (0.520)	0.510 (0.044)	0.011 (0.002)	—	—
	H-LVGGM	0.104 (0.008)	0.348 (0.042)	0.108 (0.008)	5.530 (0.674)	0.692 (0.043)	0.037 (0.015)	0.983 (0.085)	0.983 (0.084)
	OR-LVGGM	0.336 (0.042)	1.563 (0.390)	0.368 (0.043)	10.880 (2.617)	0.671 (0.037)	0.233 (0.032)	—	0.985 (0.011)
	Glasso	0.244 (0.007)	—	0.203 (0.003)	—	0.891 (0.025)	0.566 (0.037)	—	—
	OR-Glasso	0.222 (0.004)	—	0.222 (0.004)	—	0.758 (0.031)	0.385 (0.028)	—	0.995 (0.005)
	RH-GGM	0.219 (0.050)	—	0.181 (0.060)	—	0.759 (0.086)	0.286 (0.052)	0.897 (0.187)	0.869 (0.198)
	H-GGM	0.208 (0.012)	—	0.167 (0.007)	—	0.764 (0.040)	0.286 (0.032)	0.989 (0.065)	0.989 (0.061)
*p* = 100, *r* = 10	Proposed	0.119 (0.018)	0.432 (0.041)	0.129 (0.018)	10.670 (1.400)	0.648 (0.058)	0.043 (0.027)	0.868 (0.233)	0.858 (0.232)
	LVGGM	0.216 (0.006)	0.543 (0.036)	0.223 (0.005)	9.270 (0.993)	0.463 (0.038)	0.010 (0.003)	—	—
	H-LVGGM	0.120 (0.017)	0.427 (0.041)	0.129 (0.017)	10.640 (1.106)	0.649 (0.055)	0.039 (0.021)	0.904 (0.236)	0.905 (0.235)
	OR-LVGGM	0.308 (0.066)	1.322 (0.378)	0.348 (0.073)	16.127 (3.587)	0.578 (0.065)	0.176 (0.062)	—	0.986 (0.011)
	Glasso	0.268 (0.008)	—	0.210 (0.003)	—	0.894 (0.021)	0.623 (0.022)	—	—
	OR-Glasso	0.233 (0.004)	—	0.233 (0.004)	—	0.772 (0.032)	0.446 (0.033)	—	0.994 (0.007)
	RH-GGM	0.249 (0.059)	—	0.200 (0.081)	—	0.756 (0.123)	0.339 (0.081)	0.860 (0.246)	0.839 (0.246)
	H-GGM	0.239 (0.014)	—	0.185 (0.013)	—	0.771 (0.046)	0.339 (0.045)	0.898 (0.263)	0.898 (0.262)

**TABLE A2 |:** Simulation results of *π*_0_ = 0% (unbalanced setting): mean (standard deviation).

	Method	$\|\hat{S}-S{\|}_{F}/\|S{\|}_{F}$	$\|\overset{ˆ}{L}-L{\|}_{F}/\|L{\|}_{F}$	$\|\hat{\Theta }-\Theta {\|}_{F}/\|\Theta {\|}_{F}$	Rank	TPR	FPR	ARI*_c_*	ARI*_p_*
*p* = 40, *r* = 5	Proposed	0.185 (0.729)	0.364 (0.131)	0.195 (0.771)	4.970 (2.072)	0.730 (0.108)	0.047 (0.053)	0.684 (0.313)	0.687 (0.310)
	LVGGM	0.194 (0.009)	0.421 (0.064)	0.195 (0.008)	4.580 (1.165)	0.796 (0.070)	0.134 (0.055)	—	—
	H-LVGGM	0.128 (0.054)	0.402 (0.146)	0.134 (0.054)	5.120 (2.731)	0.733 (0.108)	0.064 (0.099)	0.586 (0.423)	0.586 (0.423)
	OR-LVGGM	0.239 (0.024)	0.905 (0.134)	0.240 (0.019)	6.103 (1.298)	0.694 (0.074)	0.182 (0.036)	—	0.897 (0.026)
	Glasso	0.290 (0.013)	—	0.211 (0.006)	—	0.948 (0.036)	0.783 (0.041)	—	—
	OR-Glasso	0.192 (0.007)	—	0.192 (0.007)	—	0.878 (0.047)	0.623 (0.044)	—	0.907 (0.024)
	RH-GGM	0.367 (0.887)	—	0.296 (0.950)	—	0.842 (0.122)	0.466 (0.109)	0.639 (0.352)	0.639 (0.351)
	H-GGM	0.267 (0.031)	—	0.193 (0.052)	—	0.856 (0.068)	0.471 (0.101)	0.507 (0.437)	0.507 (0.438)
*p* = 40, *r* = 10	Proposed	0.183 (0.729)	0.360 (0.121)	0.194 (0.771)	4.910 (1.688)	0.731 (0.103)	0.046 (0.050)	0.687 (0.313)	0.690 (0.309)
	LVGGM	0.194 (0.009)	0.421 (0.064)	0.195 (0.008)	4.580 (1.165)	0.796 (0.070)	0.134 (0.055)	—	—
	H-LVGGM	0.128 (0.054)	0.402 (0.146)	0.134 (0.054)	5.120 (2.731)	0.733 (0.108)	0.064 (0.099)	0.586 (0.423)	0.586 (0.423)
	OR-LVGGM	0.239 (0.024)	0.905 (0.134)	0.240 (0.019)	6.103 (1.298)	0.694 (0.074)	0.182 (0.036)	—	0.897 (0.026)
	Glasso	0.290 (0.013)	—	0.211 (0.006)	—	0.948 (0.036)	0.783 (0.041)	—	—
	OR-Glasso	0.192 (0.007)	—	0.192 (0.007)	—	0.878 (0.047)	0.623 (0.044)	—	0.907 (0.024)
	RH-GGM	0.366 (0.886)	—	0.295 (0.950)	—	0.841 (0.122)	0.466 (0.114)	0.640 (0.351)	0.640 (0.351)
	H-GGM	0.267 (0.031)	—	0.193 (0.052)	—	0.856 (0.068)	0.471 (0.101)	0.507 (0.437)	0.507 (0.438)
*p* = 100, *r* = 5	Proposed	0.127 (0.024)	0.381 (0.066)	0.130 (0.023)	5.860 (1.287)	0.670 (0.088)	0.043 (0.021)	0.674 (0.248)	0.670 (0.245)
	LVGGM	0.208 (0.006)	0.476 (0.048)	0.212 (0.006)	5.900 (0.689)	0.503 (0.047)	0.010 (0.002)	—	—
	H-LVGGM	0.117 (0.017)	0.366 (0.054)	0.120 (0.017)	5.720 (2.875)	0.702 (0.051)	0.047 (0.020)	0.814 (0.232)	0.823 (0.222)
	OR-LVGGM	0.397 (0.025)	2.144 (0.231)	0.448 (0.024)	11.213 (1.123)	0.617 (0.035)	0.192 (0.020)	—	0.966 (0.015)
	Glasso	0.243 (0.007)	—	0.202 (0.003)	—	0.893 (0.024)	0.571 (0.035)	—	—
	OR-Glasso	0.226 (0.004)	—	0.226 (0.004)	—	0.746 (0.034)	0.382 (0.031)	—	0.996 (0.005)
	RH-GGM	0.228 (0.041)	—	0.193 (0.045)	—	0.768 (0.053)	0.298 (0.061)	0.663 (0.262)	0.641 (0.267)
	H-GGM	0.213 (0.013)	—	0.175 (0.012)	—	0.767 (0.045)	0.289 (0.032)	0.807 (0.253)	0.809 (0.251)
*p* = 100, *r* = 10	Proposed	0.128 (0.017)	0.438 (0.038)	0.137 (0.016)	10.670 (1.092)	0.647 (0.048)	0.041 (0.019)	0.744 (0.243)	0.742 (0.240)
	LVGGM	0.215 (0.006)	0.542 (0.036)	0.222 (0.005)	9.200 (1.025)	0.458 (0.037)	0.010 (0.003)	—	—
	H-LVGGM	0.125 (0.017)	0.433 (0.047)	0.133 (0.016)	10.420 (1.232)	0.658 (0.047)	0.044 (0.025)	0.835 (0.236)	0.840 (0.231)
	OR-LVGGM	0.402 (0.022)	1.800 (0.143)	0.455 (0.021)	14.720 (1.196)	0.514 (0.035)	0.152 (0.013)	—	0.958 (0.018)
	Glasso	0.267 (0.009)	—	0.209 (0.004)	—	0.897 (0.023)	0.627 (0.032)	—	—
	OR-Glasso	0.237 (0.005)	—	0.237 (0.005)	—	0.762 (0.031)	0.444 (0.033)	—	0.995 (0.006)
	RH-GGM	0.319 (0.730)	—	0.274 (0.769)	—	0.758 (0.095)	0.336 (0.055)	0.732 (0.278)	0.720 (0.277)
	H-GGM	0.240 (0.013)	—	0.187 (0.011)	—	0.777 (0.035)	0.343 (0.034)	0.811 (0.261)	0.813 (0.260)

**TABLE A3 |:** Simulation results of *p* = 40 (unbalanced setting): mean (standard deviation).

	Method	$\|\hat{S}-S{\|}_{F}/\|S{\|}_{F}$	$\|\overset{ˆ}{L}-L{\|}_{F}/\|L{\|}_{F}$	$\|\hat{\Theta }-\Theta {\|}_{F}/\|\Theta {\|}_{F}$	Rank	TPR	FPR	ARI*_c_*	ARI*_o_*	ARI*_p_*
*r* = 5, *π*_0_ = 0.04	Proposed	0.134 (0.047)	0.404 (0.113)	0.144 (0.051)	4.820 (1.749)	0.722 (0.088)	0.049 (0.032)	0.576 (0.412)	0.501 (0.056)	0.528 (0.352)
	LVGGM	0.271 (0.013)	0.500 (0.059)	0.273 (0.013)	5.120 (1.725)	0.649 (0.101)	0.059 (0.058)	—	—	—
	H-LVGGM	0.248 (0.015)	0.550 (0.105)	0.256 (0.015)	4.660 (2.633)	0.721 (0.106)	0.096 (0.083)	0.081 (0.212)	—	0.085 (0.193)
	OR-LVGGM	0.244 (0.023)	0.935 (0.147)	0.248 (0.017)	6.210 (1.366)	0.689 (0.070)	0.181 (0.036)	—	—	0.831 (0.030)
	Glasso	0.335 (0.012)	—	0.275 (0.008)	—	0.944 (0.037)	0.778 (0.047)	—	—	—
	OR-Glasso	0.196 (0.007)	—	0.196 (0.007)	—	0.886 (0.043)	0.626 (0.049)	—	—	0.844 (0.028)
	RH-GGM	0.274 (0.035)	—	0.209 (0.052)	—	0.851 (0.068)	0.461 (0.097)	0.533 (0.423)	0.504 (0.057)	0.489 (0.358)
	H-GGM	0.327 (0.018)	—	0.286 (0.017)	—	0.830 (0.071)	0.445 (0.085)	0.039 (0.138)	—	0.042 (0.127)
*r* = 10, *π*_0_ = 0.04	Proposed	0.158 (0.052)	0.537 (0.167)	0.167 (0.048)	7.410 (4.080)	0.648 (0.143)	0.081 (0.088)	0.566 (0.412)	0.476 (0.057)	0.513 (0.348)
	LVGGM	0.286 (0.011)	0.576 (0.048)	0.288 (0.010)	7.220 (1.673)	0.535 (0.100)	0.029 (0.016)	—	—	—
	H-LVGGM	0.261 (0.020)	0.617 (0.119)	0.267 (0.014)	7.140 (4.411)	0.651 (0.144)	0.113 (0.087)	0.035 (0.121)	—	0.042 (0.110)
	OR-LVGGM	0.268 (0.020)	0.957 (0.088)	0.265 (0.014)	7.660 (1.552)	0.618 (0.073)	0.183 (0.044)	—	—	0.815 (0.034)
	Glasso	0.355 (0.012)	—	0.274 (0.008)	—	0.953 (0.035)	0.818 (0.043)	—	—	—
	OR-Glasso	0.204 (0.007)	—	0.204 (0.007)	—	0.886 (0.039)	0.672 (0.044)	—	—	0.834 (0.031)
	RH-GGM	0.296 (0.028)	—	0.212 (0.043)	—	0.861 (0.064)	0.533 (0.089)	0.527 (0.409)	0.476 (0.058)	0.477 (0.342)
	H-GGM	0.348 (0.016)	—	0.288 (0.014)	—	0.831 (0.064)	0.510 (0.089)	0.018 (0.075)	—	0.025 (0.067)
*r* = 5, *π*_0_ = 0.1	Proposed	0.153 (0.046)	0.444 (0.148)	0.163 (0.048)	4.390 (1.310)	0.708 (0.100)	0.062 (0.050)	0.599 (0.407)	0.446 (0.050)	0.466 (0.273)
	LVGGM	0.312 (0.011)	0.563 (0.059)	0.316 (0.010)	5.170 (1.864)	0.565 (0.088)	0.040 (0.022)	—	—	—
	H-LVGGM	0.280 (0.008)	0.574 (0.058)	0.291 (0.009)	3.920 (0.992)	0.694 (0.098)	0.091 (0.035)	0.002 (0.005)	—	0.052 (0.013)
	OR-LVGGM	0.256 (0.020)	1.011 (0.128)	0.265 (0.017)	6.607 (1.290)	0.674 (0.071)	0.174 (0.036)	—	—	0.736 (0.033)
	Glasso	0.360 (0.012)	—	0.310 (0.008)	—	0.933 (0.041)	0.763 (0.050)	—	—	—
	OR-Glasso	0.199 (0.008)	—	0.199 (0.008)	—	0.874 (0.045)	0.613 (0.049)	—	—	0.760 (0.031)
	RH-GGM	0.287 (0.032)	—	0.232 (0.047)	—	0.836 (0.067)	0.445 (0.092)	0.574 (0.417)	0.446 (0.049)	0.433 (0.276)
	H-GGM	0.344 (0.014)	—	0.308 (0.009)	—	0.800 (0.070)	0.421 (0.057)	0.003 (0.007)	—	0.053 (0.012)
*r* = 10, *π*_0_ = 0.1	Proposed	0.194 (0.049)	0.594 (0.142)	0.207 (0.044)	6.690 (3.326)	0.631 (0.136)	0.089 (0.083)	0.433 (0.429)	0.404 (0.048)	0.327 (0.273)
	LVGGM	0.318 (0.009)	0.622 (0.050)	0.323 (0.009)	7.520 (2.190)	0.496 (0.098)	0.038 (0.021)	—	—	—
	H-LVGGM	0.288 (0.009)	0.632 (0.059)	0.296 (0.009)	5.910 (2.156)	0.645 (0.120)	0.127 (0.057)	0.003 (0.006)	—	0.054 (0.014)
	OR-LVGGM	0.287 (0.021)	1.036 (0.103)	0.286 (0.017)	7.910 (1.491)	0.615 (0.066)	0.183 (0.039)	—	—	0.715 (0.032)
	Glasso	0.376 (0.011)	—	0.309 (0.007)	—	0.937 (0.036)	0.809 (0.043)	—	—	—
	OR-Glasso	0.209 (0.008)	—	0.209 (0.008)	—	0.886 (0.040)	0.666 (0.045)	—	—	0.741 (0.032)
	RH-GGM	0.321 (0.029)	—	0.265 (0.043)	—	0.838 (0.071)	0.509 (0.088)	0.345 (0.410)	0.404 (0.048)	0.268 (0.258)
	H-GGM	0.361 (0.014)	—	0.311 (0.008)	—	0.813 (0.074)	0.485 (0.064)	0.003 (0.007)	—	0.054 (0.014)

**TABLE A4 |:** Simulation results of *p* = 100 (unbalanced setting): mean (standard deviation).

	Method	$\|\hat{S}-S{\|}_{F}/\|S{\|}_{F}$	$\|\hat{L}-L{\|}_{F}/\|L{\|}_{F}$	$\|\hat{\Theta }-\Theta {\|}_{F}/\|\Theta {\|}_{F}$	Rank	TPR	FPR	ARI*_c_*	ARI*_o_*	ARI*_p_*
*r* = 5, *π*_0_ = 0.04	Proposed	0.125 (0.017)	0.382 (0.044)	0.129 (0.017)	5.680 (0.920)	0.686 (0.052)	0.051 (0.022)	0.690 (0.228)	0.525 (0.054)	0.693 (0.209)
	LVGGM	0.280 (0.008)	0.532 (0.048)	0.282 (0.008)	5.943 (0.840)	0.481 (0.047)	0.019 (0.004)	—	—	—
	H-LVGGM	0.233 (0.010)	0.470 (0.054)	0.235 (0.011)	6.356 (1.502)	0.670 (0.057)	0.074 (0.029)	0.599 (0.232)	—	0.576 (0.215)
	OR-LVGGM	0.419 (0.031)	2.254 (0.223)	0.470 (0.028)	11.203 (1.295)	0.609 (0.032)	0.196 (0.020)	—	—	0.889 (0.024)
	Glasso	0.302 (0.008)	—	0.273 (0.007)	—	0.867 (0.028)	0.540 (0.039)	—	—	—
	OR-Glasso	0.229 (0.004)	—	0.229 (0.004)	—	0.740 (0.036)	0.377 (0.034)	—	—	0.937 (0.013)
	RH-GGM	0.221 (0.015)	—	0.187 (0.013)	—	0.760 (0.040)	0.284 (0.037)	0.683 (0.237)	0.527 (0.057)	0.669 (0.220)
	H-GGM	0.287 (0.010)	—	0.263 (0.010)	—	0.748 (0.048)	0.304 (0.049)	0.564 (0.244)	—	0.537 (0.229)
*r* = 10, *π*_0_ = 0.04	Proposed	0.131 (0.016)	0.454 (0.046)	0.141 (0.015)	10.570 (1.423)	0.645 (0.047)	0.051 (0.028)	0.714 (0.229)	0.535 (0.055)	0.707 (0.209)
	LVGGM	0.283 (0.008)	0.587 (0.035)	0.288 (0.007)	9.624 (1.371)	0.438 (0.041)	0.019 (0.005)	—	—	—
	H-LVGGM	0.238 (0.013)	0.526 (0.037)	0.243 (0.014)	11.000 (1.683)	0.625 (0.060)	0.070 (0.027)	0.561 (0.267)	—	0.533 (0.245)
	OR-LVGGM	0.424 (0.021)	1.883 (0.194)	0.478 (0.025)	14.937 (1.486)	0.509 (0.040)	0.155 (0.016)	—	—	0.874 (0.024)
	Glasso	0.319 (0.009)	—	0.275 (0.007)	—	0.885 (0.021)	0.628 (0.029)	—	—	—
	OR-Glasso	0.240 (0.004)	—	0.240 (0.004)	—	0.754 (0.028)	0.433 (0.029)	—	—	0.932 (0.015)
	RH-GGM	0.246 (0.015)	—	0.198 (0.013)	—	0.770 (0.034)	0.336 (0.038)	0.706 (0.241)	0.532 (0.052)	0.681 (0.219)
	H-GGM	0.306 (0.011)	—	0.272 (0.011)	—	0.746 (0.043)	0.344 (0.044)	0.537 (0.271)	—	0.509 (0.248)
*r* = 5, *π*_0_ = 0.1	Proposed	0.133 (0.018)	0.406 (0.048)	0.139 (0.018)	5.900 (0.882)	0.671 (0.049)	0.055 (0.023)	0.623 (0.210)	0.508 (0.043)	0.591 (0.183)
	LVGGM	0.322 (0.006)	0.585 (0.049)	0.325 (0.006)	6.060 (1.135)	0.438 (0.053)	0.034 (0.007)	—	—	—
	H-LVGGM	0.292 (0.007)	0.590 (0.057)	0.298 (0.007)	5.700 (1.168)	0.629 (0.063)	0.095 (0.028)	0.129 (0.277)	—	0.169 (0.234)
	OR-LVGGM	0.465 (0.030)	2.486 (0.253)	0.518 (0.027)	11.137 (1.298)	0.600 (0.032)	0.199 (0.021)	—	—	0.770 (0.026)
	Glasso	0.339 (0.006)	—	0.316 (0.005)	—	0.847 (0.032)	0.544 (0.042)	—	—	—
	OR-Glasso	0.233 (0.004)	—	0.233 (0.004)	—	0.732 (0.036)	0.369 (0.031)	—	—	0.848 (0.022)
	RH-GGM	0.230 (0.016)	—	0.199 (0.014)	—	0.746 (0.037)	0.271 (0.030)	0.613 (0.217)	0.509 (0.042)	0.555 (0.185)
	H-GGM	0.331 (0.007)	—	0.317 (0.006)	—	0.681 (0.051)	0.273 (0.037)	0.121 (0.271)	—	0.163 (0.228)
*r* = 10, *π*_0_ = 0.1	Proposed	0.137 (0.017)	0.473 (0.040)	0.150 (0.016)	11.050 (2.022)	0.624 (0.050)	0.053 (0.023)	0.626 (0.211)	0.498 (0.044)	0.585 (0.177)
	LVGGM	0.322 (0.007)	0.630 (0.035)	0.328 (0.007)	9.830 (2.025)	0.401 (0.052)	0.034 (0.010)	—	—	—
	H-LVGGM	0.295 (0.006)	0.621 (0.035)	0.303 (0.007)	10.320 (2.079)	0.590 (0.059)	0.099 (0.031)	0.047 (0.169)	—	0.100 (0.138)
	OR-LVGGM	0.458 (0.034)	2.035 (0.167)	0.516 (0.028)	14.963 (1.674)	0.505 (0.040)	0.158 (0.017)	—	—	0.751 (0.039)
	Glasso	0.352 (0.007)	—	0.318 (0.005)	—	0.864 (0.029)	0.613 (0.041)	—	—	—
	OR-Glasso	0.244 (0.005)	—	0.244 (0.005)	—	0.746 (0.029)	0.425 (0.032)	—	—	0.841 (0.025)
	RH-GGM	0.254 (0.014)	—	0.213 (0.013)	—	0.754 (0.039)	0.324 (0.030)	0.609 (0.216)	0.496 (0.043)	0.540 (0.177)
	H-GGM	0.344 (0.008)	—	0.323 (0.006)	—	0.691 (0.042)	0.314 (0.035)	0.040 (0.161)	—	0.096 (0.132)

**TABLE A5 |:** Simulation results of *p* = 40, *r* = 5, *π*_0_ = 0, 04 under balanced setting: mean (standard deviation).

	Method	$\|\hat{S}-S{\|}_{F}/\|S{\|}_{F}$	$\|\hat{L}-L{\|}_{F}/\|L{\|}_{F}$	$\|\hat{\Theta }-\Theta {\|}_{F}/\|\Theta {\|}_{F}$	Rank	TPR	FPR	ARI*_c_*	ARI*_o_*	ARI*_p_*
*μ* = 1	Proposed	0.167 (0.009)	0.481 (0.060)	0.184 (0.011)	3.790 (0.935)	0.714 (0.081)	0.054 (0.017)	0.017 (0.052)	0.513 (0.053)	0.048 (0.046)
	LVGGM	0.258 (0.014)	0.478 (0.053)	0.260 (0.014)	4.740 (1.292)	0.692 (0.111)	0.075 (0.057)	—	—	—
	H-LVGGM	0.235 (0.011)	0.527 (0.061)	0.246 (0.013)	4.050 (1.258)	0.722 (0.087)	0.078 (0.028)	0.001 (0.002)	—	0.017 (0.017)
	OR-LVGGM	0.225 (0.018)	0.829 (0.140)	0.225 (0.017)	6.360 (1.242)	0.695 (0.067)	0.178 (0.033)	—	—	0.541 (0.046)
	Glasso	0.328 (0.013)	—	0.266 (0.009)	—	0.941 (0.044)	0.777 (0.045)	—	—	—
	OR-Glasso	0.191 (0.007)	—	0.191 (0.007)	—	0.887 (0.048)	0.630 (0.043)	—	—	0.554 (0.043)
	RH-GGM	0.287 (0.026)	—	0.242 (0.022)	—	0.840 (0.065)	0.416 (0.063)	0.010 (0.034)	0.521 (0.052)	0.041 (0.030)
	H-GGM	0.317 (0.020)	—	0.275 (0.020)	—	0.820 (0.068)	0.419 (0.061)	0.002 (0.003)	—	0.016 (0.018)
*μ* = 1.5	Proposed	0.134 (0.058)	0.417 (0.146)	0.143 (0.056)	4.810 (2.282)	0.715 (0.106)	0.063 (0.113)	0.601 (0.424)	0.503 (0.055)	0.545 (0.362)
	LVGGM	0.270 (0.014)	0.496 (0.057)	0.273 (0.014)	5.040 (1.524)	0.644 (0.103)	0.056 (0.057)	—	—	—
	H-LVGGM	0.249 (0.016)	0.550 (0.118)	0.258 (0.016)	4.680 (2.849)	0.716 (0.091)	0.092 (0.065)	0.066 (0.158)	—	0.073 (0.148)
	OR-LVGGM	0.225 (0.022)	0.802 (0.138)	0.221 (0.018)	5.763 (1.274)	0.706 (0.069)	0.194 (0.036)	—	—	0.825 (0.028)
	Glasso	0.335 (0.013)	—	0.274 (0.008)	—	0.941 (0.043)	0.778 (0.047)	—	—	—
	OR-Glasso	0.191 (0.007)	—	0.191 (0.007)	—	0.887 (0.048)	0.630 (0.043)	—	—	0.830 (0.025)
	RH-GGM	0.274 (0.035)	—	0.208 (0.052)	—	0.849 (0.069)	0.457 (0.071)	0.526 (0.445)	0.502 (0.056)	0.475 (0.375)
	H-GGM	0.326 (0.018)	—	0.285 (0.015)	—	0.831 (0.077)	0.436 (0.095)	0.039 (0.124)	—	0.047 (0.117)
*μ* = 2	Proposed	0.095 (0.028)	0.335 (0.091)	0.103 (0.023)	4.650 (0.796)	0.731 (0.087)	0.045 (0.061)	0.951 (0.147)	0.514 (0.050)	0.844 (0.128)
	LVGGM	0.279 (0.013)	0.505 (0.054)	0.282 (0.013)	5.480 (1.507)	0.612 (0.091)	0.043 (0.048)	—	—	—
	H-LVGGM	0.237 (0.021)	0.498 (0.124)	0.241 (0.021)	5.330 (3.169)	0.710 (0.112)	0.085 (0.065)	0.438 (0.254)	—	0.417 (0.239)
	OR-LVGGM	0.225 (0.018)	0.829 (0.140)	0.225 (0.017)	6.360 (1.242)	0.695 (0.067)	0.178 (0.033)	—	—	0.916 (0.017)
	Glasso	0.340 (0.012)	—	0.281 (0.008)	—	0.940 (0.046)	0.779 (0.044)	—	—	—
	OR-Glasso	0.191 (0.007)	—	0.191 (0.007)	—	0.887 (0.048)	0.630 (0.043)	—	—	0.917 (0.017)
	RH-GGM	0.249 (0.023)	—	0.166 (0.023)	—	0.852 (0.065)	0.463 (0.054)	0.950 (0.148)	0.515 (0.050)	0.835 (0.129)
	H-GGM	0.319 (0.018)	—	0.265 (0.020)	—	0.849 (0.070)	0.486 (0.087)	0.430 (0.236)	—	0.410 (0.222)
*μ* = 2.5	Proposed	0.087 (0.008)	0.317 (0.051)	0.096 (0.007)	4.740 (0.645)	0.729 (0.088)	0.034 (0.019)	0.999 (0.002)	0.522 (0.048)	0.887 (0.028)
	LVGGM	0.285 (0.011)	0.509 (0.048)	0.288 (0.012)	5.960 (1.271)	0.587 (0.094)	0.033 (0.031)	—	—	—
	H-LVGGM	0.218 (0.011)	0.424 (0.051)	0.221 (0.011)	4.930 (1.047)	0.732 (0.080)	0.073 (0.031)	0.963 (0.119)	—	0.903 (0.109)
	OR-LVGGM	0.225 (0.018)	0.829 (0.140)	0.225 (0.017)	6.360 (1.242)	0.695 (0.067)	0.178 (0.033)	—	—	0.935 (0.013)
	Glasso	0.343 (0.012)	—	0.285 (0.008)	—	0.940 (0.046)	0.781 (0.044)	—	—	—
	OR-Glasso	0.191 (0.007)	—	0.191 (0.007)	—	0.887 (0.048)	0.630 (0.043)	—	—	0.935 (0.013)
	RH-GGM	0.245 (0.020)	—	0.161 (0.009)	—	0.848 (0.068)	0.460 (0.051)	0.998 (0.002)	0.523 (0.048)	0.879 (0.029)
	H-GGM	0.307 (0.016)	—	0.245 (0.011)	—	0.849 (0.065)	0.490 (0.055)	0.942 (0.148)	—	0.882 (0.137)

**TABLE A6 |:** Simulation results of *p* = 100, *r* = 5, *π*_0_ = 0, 04 under balanced setting: mean (standard deviation).

	Method	$\|\hat{S}-S{\|}_{F}/\|S{\|}_{F}$	$\|\hat{L}-L{\|}_{F}/\|L{\|}_{F}$	$\|\hat{\Theta }-\Theta {\|}_{F}/\|\Theta {\|}_{F}$	Rank	TPR	FPR	ARI*_c_*	ARI*_o_*	ARI*_p_*
*μ* = 1	Proposed	0.155 (0.016)	0.480 (0.078)	0.165 (0.018)	6.120 (1.887)	0.655 (0.076)	0.048 (0.024)	0.136 (0.296)	0.504 (0.065)	0.159 (0.276)
	LVGGM	0.277 (0.008)	0.530 (0.047)	0.279 (0.008)	5.660 (0.691)	0.482 (0.047)	0.018 (0.004)	—	—	—
	H-LVGGM	0.242 (0.009)	0.510 (0.052)	0.246 (0.009)	6.278 (1.281)	0.656 (0.059)	0.066 (0.025)	0.002 (0.003)	—	0.029 (0.012)
	OR-LVGGM	0.360 (0.039)	1.789 (0.358)	0.400 (0.038)	11.687 (2.200)	0.659 (0.037)	0.232 (0.028)	—	—	0.747 (0.038)
	Glasso	0.299 (0.008)	—	0.269 (0.007)	—	0.872 (0.028)	0.544 (0.044)	—	—	—
	OR-Glasso	0.224 (0.004)	—	0.224 (0.004)	—	0.754 (0.035)	0.379 (0.030)	—	—	0.833 (0.026)
	RH-GGM	0.230 (0.011)	—	0.210 (0.008)	—	0.749 (0.039)	0.259 (0.043)	0.062 (0.211)	0.519 (0.061)	0.090 (0.197)
	H-GGM	0.290 (0.009)	—	0.272 (0.007)	—	0.722 (0.043)	0.266 (0.032)	0.001 (0.002)	—	0.028 (0.014)
*μ* = 1.5	Proposed	0.111 (0.015)	0.370 (0.051)	0.116 (0.015)	5.600 (0.778)	0.687 (0.049)	0.045 (0.020)	0.872 (0.162)	0.541 (0.056)	0.854 (0.150)
	LVGGM	0.280 (0.008)	0.534 (0.044)	0.282 (0.008)	6.057 (0.807)	0.480 (0.047)	0.019 (0.004)	—	—	—
	H-LVGGM	0.239 (0.012)	0.505 (0.060)	0.243 (0.013)	6.124 (1.423)	0.662 (0.060)	0.075 (0.032)	0.387 (0.236)	—	0.370 (0.223)
	OR-LVGGM	0.377 (0.059)	1.940 (0.510)	0.422 (0.065)	11.966 (2.226)	0.649 (0.045)	0.229 (0.029)	—	—	0.927 (0.022)
	Glasso	0.303 (0.008)	—	0.274 (0.007)	—	0.870 (0.031)	0.543 (0.045)	—	—	—
	OR-Glasso	0.226 (0.006)	—	0.226 (0.006)	—	0.749 (0.037)	0.376 (0.031)	—	—	0.936 (0.016)
	RH-GGM	0.214 (0.013)	—	0.177 (0.010)	—	0.753 (0.042)	0.273 (0.032)	0.877 (0.156)	0.543 (0.055)	0.842 (0.146)
	H-GGM	0.289 (0.011)	—	0.269 (0.012)	—	0.729 (0.043)	0.280 (0.039)	0.331 (0.241)	—	0.314 (0.227)
*μ* = 2	Proposed	0.107 (0.005)	0.363 (0.043)	0.112 (0.005)	5.580 (0.867)	0.686 (0.042)	0.042 (0.016)	0.918 (0.017)	0.553 (0.052)	0.900 (0.025)
	LVGGM	0.282 (0.007)	0.535 (0.047)	0.284 (0.007)	6.212 (0.742)	0.482 (0.047)	0.021 (0.004)	—	—	—
	H-LVGGM	0.231 (0.008)	0.455 (0.038)	0.233 (0.009)	6.176 (1.071)	0.681 (0.051)	0.073 (0.025)	0.944 (0.152)	—	0.890 (0.141)
	OR-LVGGM	0.362 (0.041)	1.802 (0.372)	0.403 (0.040)	11.663 (2.248)	0.661 (0.036)	0.233 (0.029)	—	—	0.941 (0.013)
	Glasso	0.303 (0.008)	—	0.274 (0.006)	—	0.876 (0.026)	0.551 (0.045)	—	—	—
	OR-Glasso	0.224 (0.004)	—	0.224 (0.004)	—	0.757 (0.034)	0.380 (0.031)	—	—	0.941 (0.012)
	RH-GGM	0.211 (0.012)	—	0.174 (0.005)	—	0.755 (0.037)	0.273 (0.029)	0.918 (0.018)	0.553 (0.052)	0.886 (0.027)
	H-GGM	0.286 (0.010)	—	0.261 (0.008)	—	0.734 (0.038)	0.287 (0.030)	0.927 (0.169)	—	0.874 (0.159)
*μ* = 2.5	Proposed	0.107 (0.005)	0.363 (0.043)	0.112 (0.005)	5.510 (0.674)	0.689 (0.043)	0.043 (0.017)	0.921 (0.016)	0.562 (0.050)	0.902 (0.025)
	LVGGM	0.283 (0.007)	0.538 (0.047)	0.286 (0.007)	6.411 (0.707)	0.476 (0.049)	0.022 (0.004)	—	—	—
	H-LVGGM	0.230 (0.009)	0.454 (0.038)	0.232 (0.009)	6.326 (1.143)	0.676 (0.051)	0.070 (0.024)	1.000 (0.000)	—	0.940 (0.012)
	OR-LVGGM	0.360 (0.040)	1.790 (0.363)	0.401 (0.039)	11.649 (2.184)	0.659 (0.036)	0.232 (0.029)	—	—	0.940 (0.012)
	Glasso	0.305 (0.008)	—	0.276 (0.006)	—	0.874 (0.027)	0.552 (0.046)	—	—	—
	OR-Glasso	0.224 (0.004)	—	0.224 (0.004)	—	0.754 (0.035)	0.380 (0.030)	—	—	0.940 (0.012)
	RH-GGM	0.211 (0.011)	—	0.173 (0.005)	—	0.757 (0.036)	0.274 (0.027)	0.921 (0.017)	0.563 (0.050)	0.889 (0.026)
	H-GGM	0.286 (0.010)	—	0.261 (0.008)	—	0.730 (0.038)	0.287 (0.029)	1.000 (0.000)	—	0.940 (0.012)

## Appendix B Additional Data Analysis Results

**TABLE A7 |:** Cross-table of sample counts: clustering results versus CLAUDIN labels.

	Subgroup 1	Subgroup 2	Subgroup 3	Subgroup 4	Subgroup 5	Subgroup 6	Subgroup 7	Subgroup 8	Subgroup 9
Basal-like	8	15	1	3	3	1	66	7	3
Claudin-low	4	18	8	4	40	25	50	8	0
Her2	18	113	12	5	4	0	0	21	23
LumA	1	18	63	221	286	50	0	16	22
LumB	0	37	220	80	29	0	0	85	3
Normal-like	4	10	10	12	50	37	2	5	4
NC	0	0	0	1	3	0	0	0	1
Sum	35	211	314	326	415	113	118	142	56

**TABLE A8 |:** Cross-table of sample counts: clustering results versus INTCLUST labels.

	Subgroup 1	Subgroup 2	Subgroup 3	Subgroup 4	Subgroup 5	Subgroup 6	Subgroup 7	Subgroup 8	Subgroup 9
1	0	11	45	10	14	1	1	40	3
2	1	2	34	9	14	0	0	9	1
3	4	2	25	73	130	34	0	9	3
4ER−	15	8	2	1	6	4	12	2	4
4ER+	4	11	28	31	82	59	2	11	5
5	0	157	1	1	2	0	0	3	0
6	0	0	34	5	16	0	0	25	3
7	2	1	34	57	57	2	0	10	16
8	0	0	59	119	73	13	0	7	17
9	2	16	47	17	19	0	5	17	4
10	7	3	5	3	2	0	98	9	0
Sum	35	211	314	326	415	113	118	142	56
